# Investigating short windows of interbrain synchrony: A step toward fNIRS-based hyperfeedback

**DOI:** 10.1162/IMAG.a.43

**Published:** 2025-06-17

**Authors:** Kathrin Kostorz, Trinh Nguyen, Yafeng Pan, Filip Melinscak, David Steyrl, Yi Hu, Bettina Sorger, Stefanie Hoehl, Frank Scharnowski

**Affiliations:** Department of Cognition, Emotion, and Methods in Psychology, Faculty of Psychology, University of Vienna, Vienna, Austria; Department of Developmental and Educational Psychology, Faculty of Psychology, University of Vienna, Vienna, Austria; Neuroscience of Perception and Action Lab, Italian Institute of Technology, Rome, Italy; Department of Developmental and Biological Psychology, Heidelberg University, Heidelberg, Germany; Department of Psychology and Behavioral Sciences, Zhejiang University, Hangzhou, China; The State Key Lab of Brain-Machine Intelligence, Zhejiang University, Hangzhou, China; Shanghai Key Laboratory of Mental Health and Psychological Crisis Intervention, School of Psychology and Cognitive Science, East China Normal University, Shanghai, China; Department of Cognitive Neuroscience, Faculty of Psychology and Neuroscience, Maastricht University, Maastricht, The Netherlands

**Keywords:** hyperscanning, neurofeedback, fNIRS, interbrain synchrony, interpersonal neural synchrony, hyperfeedback

## Abstract

Social interaction is of fundamental importance to humans. Prior research has highlighted the link between interbrain synchrony and positive outcomes in human social interaction. Neurofeedback is an established method to train one’s brain activity and might offer a possibility to increase interbrain synchrony, too. Consequently, it would be advantageous to determine the feasibility of creating a neurofeedback system for enhancing interbrain synchrony to benefit human interaction. One vital step toward developing a neurofeedback setup is to determine whether the target metric can be determined in relatively short time windows. In this study, we investigated whether the most widely employed metric for interbrain synchrony, wavelet transform coherence, can be assessed accurately in short time windows using functional near-infrared spectroscopy (fNIRS), which is recognized for its mobility and ecological suitability for interactive research. To this end, we have undertaken a comprehensive approach where we created artificial data of different noise levels of a dyadic interaction and re-processed two human-interaction datasets. For both artificial and in vivo data, we computed short windows of interbrain synchrony of varying size and assessed significance at each window size. Our findings indicate that relatively short windows of wavelet transform coherence of integration durations of about 1 minute are feasible. This would align well with the methodology of an intermittent neurofeedback procedure. Our investigation lays a foundational step toward an fNIRS-based system to measure interbrain synchrony in real time and provide participants with information about their interbrain synchrony. This advancement is crucial for the future development of a neurofeedback training system tailored to enhance interbrain synchrony to potentially benefit human interaction.

## 1 Introduction

Social interaction is vital not only for our survival but also for our physical and mental health (Cohen, 2004). Our interactions range from verbal to nonverbal communication, and they promote cooperating with each other, bonding with each other, as well as learning from one another. Impaired social interaction abilities, in contrast, are detrimental to us and have been associated with, for example, learning disorders (Kavale & Forness, 1996) or psychiatric disorders (Schilbach, 2016).

While traditionally, social neuroscience research has focused on single subjects reacting to social stimuli, in recent years, the focus has shifted toward investigating subjects in direct interaction (e.g.,Dumas, 2011;Hari & Kujala, 2009;Schilbach et al., 2013). This so-called second-person neuroscience provides not only increased ecological validity but has also revealed distinct neural signatures associated with direct interactions (Redcay & Schilbach, 2019). It is thought that the neural signals of each interacting agent become coupled and aligned through the agents’ reciprocal behavior (Dumas, 2011;Hasson et al., 2012;Hasson & Frith, 2016;Kingsbury & Hong, 2020).

To investigate how two brain signals are coupled during social interaction, interbrain synchrony (IBS) is typically determined (e.g.,Czeszumski et al., 2022). IBS signifies the temporal co-occurrence and alignment of neuronal signals, thus indicating that the signals are temporally related. Using the framework of active inference, it has been suggested that two brains predict each other during interaction, and synchronization emerges naturally (Friston & Frith, 2015a,2015b;Koban et al., 2017). According to this framework, IBS facilitates mutual prediction by minimizing the social prediction error (Friston & Frith, 2015b;Hamilton, 2020;Hoehl et al., 2021;Kingsbury et al., 2019;Koban et al., 2017;Pan et al., 2023;Shamay-Tsoory et al., 2019).

To measure IBS during an interaction, it is necessary to image two brains simultaneously, that is, to perform so-called hyperscanning (Montague et al., 2002; seeCzeszumski et al., 2020for an overview). Hyperscanning has by now been established in all major neuroimaging technologies, including functional near-infrared spectroscopy (fNIRS) (e.g.,Babiloni & Astolfi, 2014). A major advantage of fNIRS for hyperscanning is that it is mobile and can be applied in nearly all natural environments, especially in comparison with functional magnetic resonance imaging (fMRI). Further, participants are able to sit, stand, or move in their natural upright position while undergoing fNIRS neuroimaging. Two or more individuals can face each other directly and talk or interact manually with each other in a naturalistic way. While this is also possible with electroencephalography (EEG), fNIRS has the added advantage of increased spatial resolution: The spatial resolution of 1–3 cm in fNIRS is better than 5–9 cm in EEG, and due to the optic technology, the signal received is localized to the light pathway (Ferrari & Quaresima, 2012;Pinti et al., 2020). Combined with considerably lower susceptibility to motion artifacts, fNIRS is thought to be the go-to method for mobile imaging (Klein et al., 2024;Kohl et al., 2020;Pinti et al., 2020).

Using fNIRS, IBS has been tested in a range of hyperscanning studies investigating different aspects of social interaction, both in adults and in adult–child interaction (e.g.,Nguyen et al., 2020;Reindl et al., 2018). This has been investigated mainly in dyadic settings, that is, two persons interacting, but also in larger groups, for example, 3-on-3 person competition (J. Yang et al., 2020). The types of social interactions investigated with fNIRS and IBS range from cooperative tapping experiments (e.g.,Cui et al., 2012;Funane et al., 2011) and creative problem solving (e.g.,Xue et al., 2018) to teaching (e.g.,Nozawa et al., 2019;Pan et al., 2017;Zheng et al., 2018), and counselling (Y. Zhang et al., 2018); see alsoCzeszumski et al. (2022)for a recent meta-analysis. Specifically, the strength of IBS as measured by fNIRS has been associated with cooperative problem-solving success (e.g.,Cui et al., 2012;Nguyen et al., 2020;Pan et al., 2017;Reindl et al., 2018) and prosociality during cooperation (Hu et al., 2017), as well as learning outcomes in teaching settings (e.g.,Pan et al., 2018,^2020^;Zhang et al., 2022). Taken together, these studies suggest that higher IBS is overall associated with better interactive performance, both with the information transfer between individuals during interactions and with associated social motivations.

In these studies, IBS is typically investigated offline and post hoc as an average over large experimental durations. This, however, falls short of the potentially dynamic nature of IBS, which may fluctuate over the course of an interaction, especially in naturalistic scenarios (Gordon et al., 2025). Being able to calculate short-time IBS may give additional insights into dynamic synchrony processes and their relation to momentary behavioral measures, as well as outcome measures such as learning or bonding. Moreover, if short-time IBS calculations are possible, this poses a first step toward a real-time IBS detection system. As such, momentary IBS states could be used in brain–computer interfaces or neurofeedback systems.

Neurofeedback is a tool to help subjects train their neural state to enhance it. Given the reported positive associations between IBS and human interaction, it is reasonable to hypothesize that enhancing IBS may lead to valuable improvements in human interaction. This may be especially the case where human interaction is impaired, as is the case in, for example, certain psychiatric conditions (Schilbach, 2016). Especially in psychiatric or neurodivergent conditions such as social anxiety disorder or autism spectrum disorder, interpersonal synchrony can be impaired, and therapeutic interventions based on enhancing synchrony are proposed (Konrad et al., 2024).

Neurofeedback is an established method which provides participants with information about their neural state with the goal of aiding them in regulating it (e.g.,Birbaumer et al., 2013;Sitaram et al., 2017). It is established in EEG and fMRI and has also recently gained traction in the domain of fNIRS (Godet et al., 2023;Kober et al., 2018;Kohl et al., 2020,^2023^;Soekadar et al., 2021). During a neurofeedback session, a participant’s neural state is measured in real time, and information about this state is then fed back to the participant in the form of, for example, a visual thermometer or an auditory tone. With the help of feedback, participants become able to monitor their own brain states and can learn to self-regulate certain aspects (e.g., activation level, connectivity strength) of their brain activity (Paret et al., 2019). These learning processes are thought to be mediated by reinforcement learning/operant conditioning or motor skill learning processes (Enriquez-Geppert et al., 2017;Lubianiker et al., 2022;Sitaram et al., 2017). It should be noted that methodological challenges and pitfalls remain. These range from technical challenges of real-time signal processing to assessing non-response, finding appropriate control conditions for clinical trials, and optimizing intervention designs (Enriquez-Geppert et al., 2017;Thibault et al., 2016,^2018^). Nevertheless, efforts are ongoing to improve neurofeedback designs (Sorger et al., 2019). Learning self-regulation through neurofeedback training has been demonstrated to change the behavior associated with the trained brain states. Neurofeedback offers the advantage of a non-invasive brain-based intervention to modify behavior in a performance-training, experimental, or clinical context (Enriquez-Geppert et al., 2017;Sitaram et al., 2017). It has been shown to improve clinical symptoms in psychiatric and neurological patients (e.g.,Haugg et al., 2021;Sitaram et al., 2017). FNIRS-based neurofeedback may be especially suitable for mobile, real-world interventions (Klein et al., 2024).

Neurofeedback allows participants to gain access to their brain state. In the case of two interacting participants, this would mean receiving additional information about both participants’ brain states or the direct relation of both brain states, as would be the case in IBS neurofeedback. Normally, during an interaction, one person’s brain state can only be communicated to another person behaviorally, that is, through speech or actions (e.g.,Dumas, 2011;Kingsbury & Hong, 2020). By adding neurofeedback to an interaction, the natural information transfer between two brains through behavior can be enhanced with information about the interacting brain states.

Given the potential benefits of providing neurofeedback of IBS, it has been proposed and called for repeatedly (Gvirts Provolovski & Perlmutter, 2021;Holroyd, 2022;Liu et al., 2019;Moreau & Dumas, 2021;Saul et al., 2022;Vanutelli & Lucchiari, 2022). However, it has only been sparsely attempted so far. Using EEG, pioneering work has been done in artistic contexts, where participants were visually fed back information about their brain state during interaction (Dikker et al., 2019,^2021^;Kovacevic et al., 2015). Recently,Müller et al. (2021)have demonstrated controllability over theta band synchrony in EEG, and an interface for EEG hyperfeedback has been presented (Chen et al., 2021). In animals, a proof-of-principle study demonstrated control of IBS in pigeons using invasive electrophysiological recordings (L. Yang et al., 2020). Using fNIRS in humans, the concept of cross-brain neurofeedback has been described (Duan et al., 2013); however, in this proof-of-principle study, two subjects were instructed to activate their brain activity higher than their partner, which is different from controlling IBS directly.

Although the scientific and possible clinical benefits of IBS neurofeedback are accepted (Konrad et al., 2024;Saul et al., 2022), there is yet no working implementation in the domain of fNIRS. This is likely due to methodological challenges, including modest signal quality, small effect sizes, and the ongoing refinement of real-time signal processing, which has yet to reach standardization (Kohl et al., 2020).

Here, we aim to take the first step toward hyperfeedback, meaning toward constructing an fNIRS-neurofeedback setup based on IBS. We aim to demonstrate that the to-be-fed-back IBS signal, which carries the neural information, can be calculated meaningfully in short time intervals. For any kind of feedback signal, it is vital that the metric can be determined in short time intervals. This means that the IBS signal needs to be strong and robust enough in short intervals to still be detectable. With these prerequisites, we ensure that short intervals of IBS carry meaningful information on the single-dyad level. We test these prerequisites of IBS neurofeedback by simulating and re-analyzing dyadic fNIRS IBS data.

A crucial step is the selection of the IBS metric. Currently, wavelet transform coherence (WTC) is by far the most used metric for investigating interbrain synchrony in fNIRS experiments (Czeszumski et al., 2022;Hakim et al., 2023). The wavelet transform splits a signal in time and frequency/period bands with the use of small, localized waves, the wavelets. While different wavelet shapes have been created for different signal processing use cases, the versatile Morlet wavelet is most commonly used in fNIRS IBS analysis. IBS is calculated via the coherence of two wavelet-transformed signals. WTC identifies how synchronized two signals are across different periods and time units. While other metrics like the Pearson correlation are also in use for real-time signal analysis, especially outside the field of fNIRS, these other IBS methods often do not lead to the same synchrony values in the same experimental contexts (Hakim et al., 2023). To assure comparability to the current literature, we will, therefore, investigate short windows of WTC and see whether they still provide a significant contrast in our simulations and re-analyzed data sets.

## 2 Methods

### 2.1 Overview

To assess the feasibility of short windows of interbrain synchrony, we performed simulations as well as a re-analysis of two existing data sets, seeFigure 1. For the simulations, we adopted a hemodynamic task model of interacting persons. Additionally, we re-analyzed two existing data sets from interacting persons. To assess short windows of interbrain synchrony, we took windows of varying sizes from the task conditions and calculated the WTC. We took the first rest condition as one baseline window. We then tested whether the difference between the windowed task WTC and the baseline WTC is significant, which would be indicative of a meaningful contrast, under the null hypothesis that there is no difference between the WTC task windows and the baseline.

**Fig. 1.**
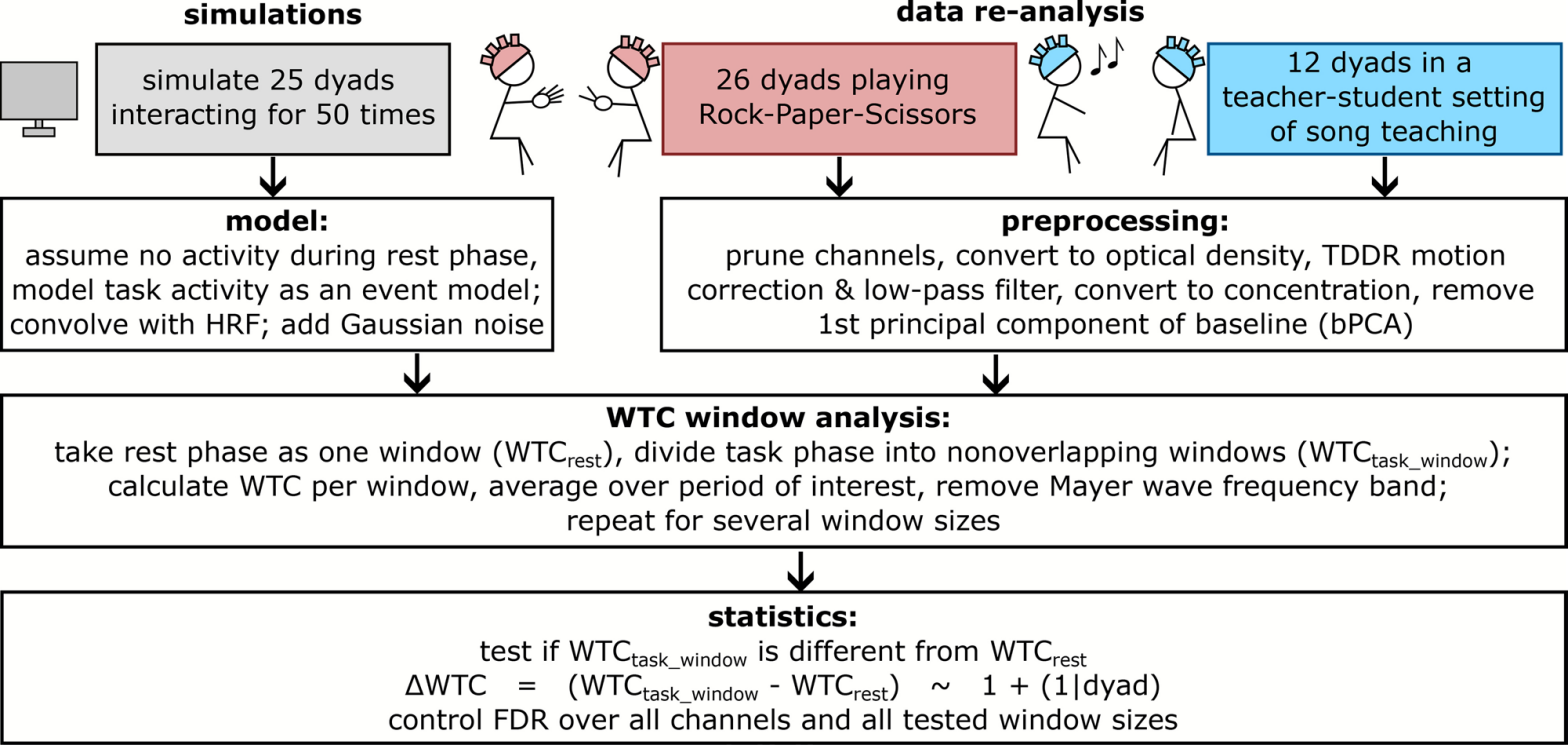
Processing pipeline for this manuscript. We simulated artificial and re-analyzed existing fNIRS data of interacting dyads. Of these data, the same windowed WTC analysis and subsequent statistics were performed. The significance of short WTC windows was assessed for all data sets.

### 2.2 Simulations

**Fig. 2.**
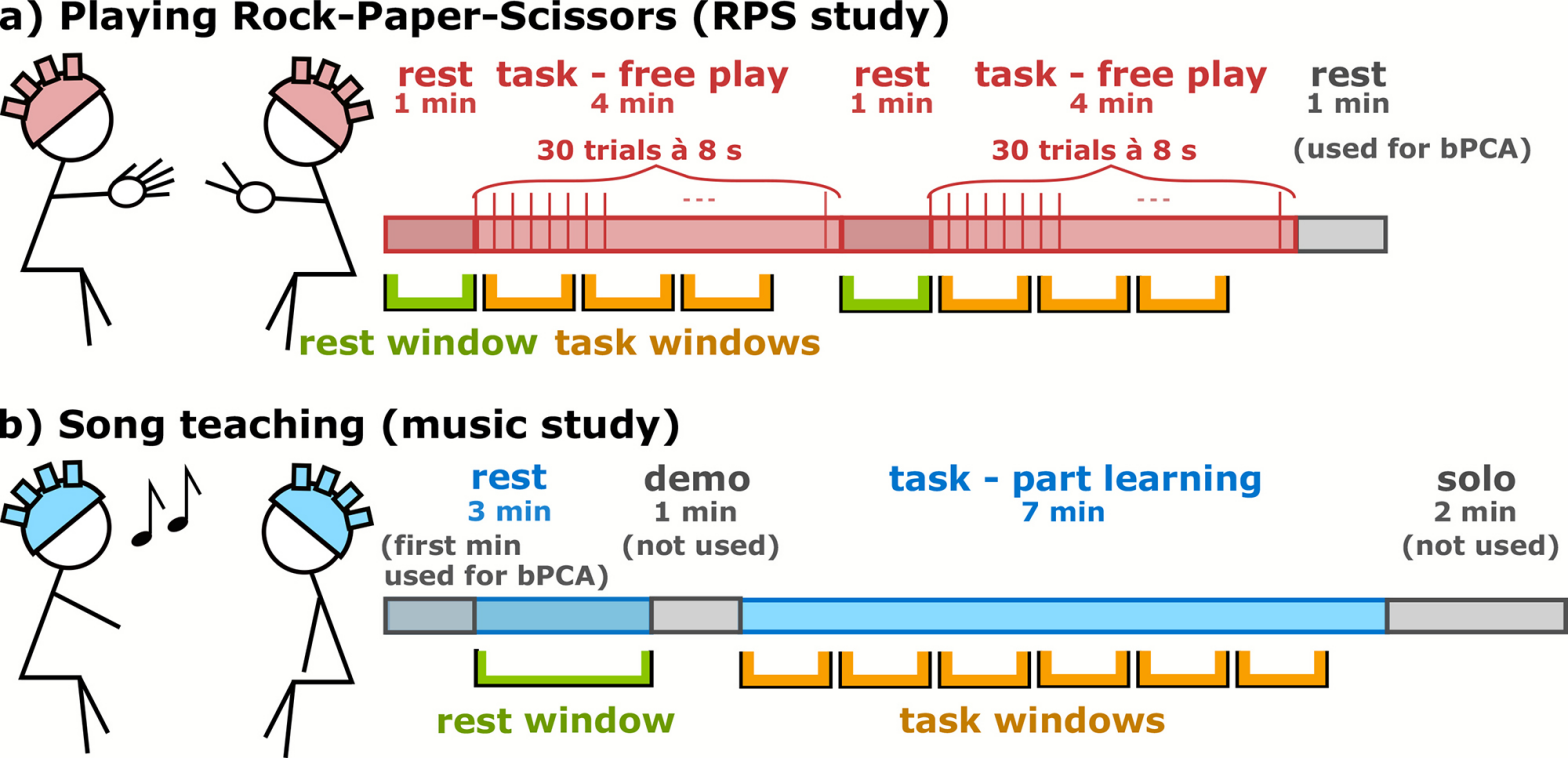
Experimental designs of the data sets and window selection for data re-analysis. (a) For the RPS study, subjects played Rock-Paper-Scissors with a timed initiation of trial every 8 seconds (free play condition, seeKayhan et al., 2022) in two blocks of a rest phase followed by a task phase. For the data re-analysis, we used both experimental blocks. We used the complete rest phase as a WTC baseline window (green window) and non-overlapping windows of predefined varying sizes (orange windows) for the task phase. (b) For the music study, a teacher taught a song to a student partwise (part learning condition, seePan et al., 2018). For the data re-analysis, we used 2 minutes of the rest phase as a WTC baseline window (green window) and non-overlapping windows of predefined varying sizes (orange windows) for the task phase. For both data sets, 1 minute of the rest phases was used for the baseline PCA (bPCA) during preprocessing to assess the extracerebral systemic activity pattern.

To gain an understanding of the theoretical behavior of the data, we ran simulations that mimicked the design of one of the data sets, the Rock-Paper-Scissors data set (Fig. 2a;Kayhan et al., 2022). We modeled every simulated time course of a person as an activation design which consisted of a 60-second rest phase where no activity was assumed, followed by a task phase of 30 events with an intertrial interval of 8 seconds, followed by another 60-second rest phase afterward. This model was convolved with SPM12’s hemodynamic response function (https://www.fil.ion.ucl.ac.uk/spm/software/spm12/). Gaussian noise was added afterward to create the artificial signal of this one subject. The artificial time course of the second person of the dyad was created in the same way. For each simulated dyad, windowed WTC calculations were then carried out. The WTC calculations were performed via the same routine as for the data re-analysis, seeSection 2.4. To investigate the effect of different signal-to-noise ratios (SNR) on the results, we simulated 25 dyads with Gaussian noise levels between 1.90 dB and -12.08 dB. At each SNR level, the simulations were run for 50 iterations.

### 2.3 Re-analysis of existing data

To assess the performance of the analysis pipeline on real data, we re-analyzed the original data from two existing hyperscanning studies (Figs. 1and2). The original data collection was carried out under the ethics approval of the Medical Faculty of the Leipzig University (RPS study) and East China Normal University (music study). The original ethics applications included consent for secondary data analysis, as carried out here.

#### 2.3.1 Description of the data sets

One of the studies, the RPS study, assessed the synchrony between two participants playing Rock-Paper-Scissors (Kayhan et al., 2022). In the study, dyads had to play in different conditions where they either had to predict each other’s actions or play freely. For the analysis in this manuscript, we only used the free play condition as it resulted in the biggest IBS contrast to the resting phase in the original analysis. The dyad specificity of this condition was also shown in the original manuscript by comparing real and random pairings. In this condition, the dyads had to play Rock-Paper-Scissors as they normally do receiving a prompt to do so every 8 seconds. During this condition, a rest phase of 60 seconds was followed by a task phase of 4 minutes twice (seeFig. 2a). For this analysis, complete data from 26 dyads were available and used. For the original study, 32 right-handed, same-sex dyads were recruited (20 female, M = 23.8 years, SD = 2.8 years, age range 18–30 years).

Signals were measured using an NIRScout 16–16 (NIRx Medizintechnik GmbH, Germany) optical topography system using wavelengths of 760 and 850 nm and a sampling rate of 7.81 Hz. For each participant, 8 sources and 8 detectors were used, resulting in 16 channels on four 2 × 2 probe sets. Two probe sets were placed over the left and right dorsolateral prefrontal cortex (DLPFC) surrounding EEG 10–20 locations F3 and F4, the other two probe sets were placed over the left and right temporo-parietal junction (TPJ) surrounding CP5 and CP6.

The second study, the music study, investigated the IBS between teacher and learner while learning a song (Pan et al., 2018). Here, one half of the dyads of teacher and learner were to learn a song as a whole, and the other half were to learn a song part wise, following musical phrases. For this analysis, we only used the “part-learning” condition, as it showed higher IBS compared with the rest phase than the other condition, as well as a correlation with subsequent song performance. Dyad specificity was also assessed in the original manuscript by investigating random pairings. For this analysis, we used a rest phase of 3 minutes and a task phase of 7 minutes (seeFig. 2b). This analysis was performed on the data of 12 dyads of the part-learning condition. For the original study, 24 students (age 20.58 ± 2.15 years) and 1 music instructor (age 22 years) were recruited. All participants were female and right-handed.

Signals were obtained using an ETG-7100 optical topography system (Hitachi Medical Corporation, Japan) using wavelengths of 695 and 830 nm and a sampling rate of 10 Hz. Two probe sets (3 × 5) were used for each participant’s left and right fronto-temporal-parietal regions.

In this manuscript, we present the results based on the chromophore used in the original analysis, that is, HbO for the music study and HbR for the RPS study. The results of the other chromophore can be found in the Supplementary Information (Supplementary Figs. S4 and S5).

#### 2.3.2 Preprocessing

To ensure comparability, we preprocessed both data sets in the same way. We opted for a routine that is “real-time-ready,” meaning it could be adapted to a real-time scenario, following the suggestions ofKlein (2024). Specifically, we used the functions of the Homer 2 toolbox (https://homer-fnirs.org/) integrated into MATLAB scripts. We first removed corrupt channels using “enPruneChannels” (SD range 20–45 mm; SNRthresh = 2; dRange 0.01–4 (music data), 0.01–2.5 (RPS data)). For the music data set, given the number of 12 dyads, if the same channel was corrupted for 3 or more dyads, we have removed the channel from the analysis completely. This has led to the exclusion of 7 channels (out of 42) for the music data set. We then converted the signal to optical density using “hmrIntensity2OD.” Motion correction was performed using the Temporal Derivative Distribution Repair (TDDR) algorithm (Fishburn et al., 2019). As the TDDR algorithm already requires inherent low-pass filtering, we combined motion correction and filtering into one step. We adapted the “hmrMotionCorrectTDDR” function slightly by changing the cutoff to 0.2 Hz instead of the default 0.5 Hz to eliminate frequencies related to respiration artifacts. While previously filtered-out high frequencies can be added again after the motion correction routine (Fishburn et al., 2019),we removed this option from the code to make full use of the inherent low-pass filter. We then converted the data to changes in concentration using “hmrOD2Conc” (partial pathlength factor 6). To account for systemic extracerebral artifacts (Tachtsidis & Scholkmann, 2016), we performed a baseline principal component analysis (bPCA), which resulted in the best systemic artifact removal if short distance channels are unavailable (Santosa et al., 2020), as is the case in the two data sets used here. The bPCA estimates the spatial profile of the noise based on a separate baseline (Y. Zhang et al., 2005). To perform the bPCA, we adapted the function “hmrPCAFilter” by feeding in separate baseline data. The spatial noise components were calculated from unused baseline data, meaning rest phase data that were not used for the synchrony investigation. We then removed the projection of the first noise component onto the investigation data from the investigation data. For the RPS data set, the last rest phase of 1 minute was used as a baseline for the bPCA, for the music data set, the 1st minute of the 3-minute rest phase was used (see alsoFig. 2).

### 2.4 Wavelet transform coherence analysis

For calculating the coherence in a pseudo-real-time fashion, meaning calculating it as it would be necessary in a real-time situation, we adapted the currently used WTC approaches. Wavelet transform coherence was estimated using the “wcoherence” function from the MATLAB wavelet toolbox, which follows the work ofGrinsted et al. (2004). We used the default setting of Morlet/Gabor wavelets and averaged over a pre-defined period band of interest. For all analyses in this manuscript, we have chosen a period band of interest of 6 to 14 seconds, which corresponds to the upper and lower period boundaries utilized in the original analyses of the re-analyzed studies of this manuscript. To avoid detecting a possible confounding synchronization of Mayer waves, we censored periods between 9 and 11 seconds of the WTC analysis, which effectively led to periods of 6–9 and 11–14 seconds we averaged over. We have chosen 14 “VoicesPerOctave” (14 subbands/wavelets) instead of the default 12 to achieve the best coverage of the 6–14 seconds period band of interest.

**Fig. 3.**
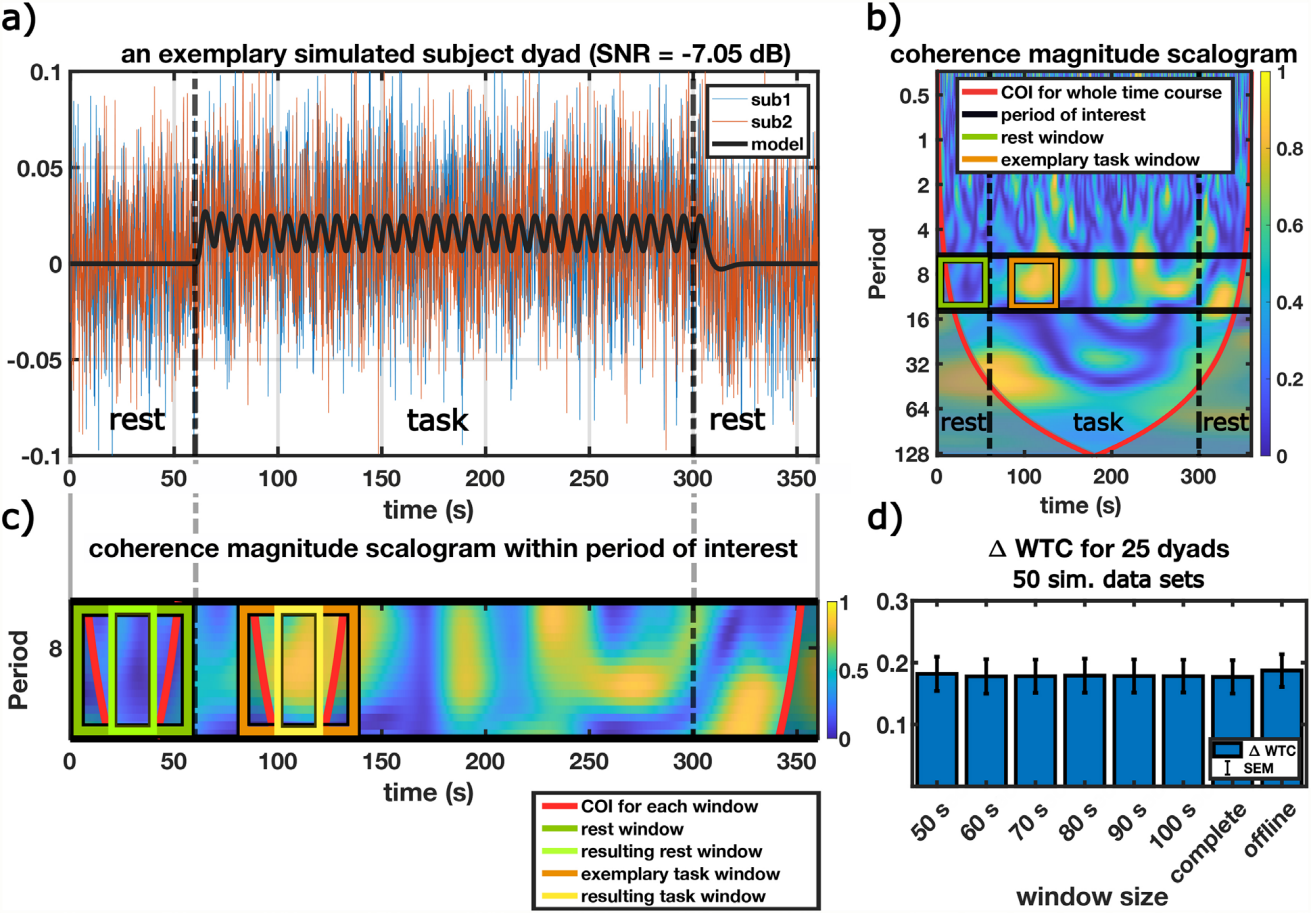
Simulations of dyads for a high noise level (SNR = -7.05 dB). (a) An exemplary simulated dyad with a model of zero activity for the rest phase and event-related activity for the task phase plus a high level of added Gaussian noise. (b) The coherence magnitude scalogram for the dyad depicted in (a). The analysis windows are marked in green (rest phase window) and orange (exemplary task window). (c) Close-up of the magnitude scalogram of (b) for the period band of interest (black lines). The relationship between a window and the COI is shown: for the exemplary task window (orange), the COI is plotted (red), and the resulting smaller window (yellow) after considering the COI. The same is plotted for the rest phase baseline window (dark green for the raw window and light green for the resulting smaller window). (d) Difference in WTC (WTC_task_window_– WTC_rest_) calculated for the 25 simulated dyads, rerun for 50 iterations. The bar depicts the average intercept of the mixed-effects model over these 50 iterations, the error bars are the standard errors of the mean of the intercept averaged over 50 iterations. The data correspond to the SNR = -7.05 dB entries ofTable 1.

For a real-time experiment, it is essential to determine how long data need to be integrated before feedback can be provided. Here, this means how many time points need to be aggregated together (the “window of integration”) to feed a meaningful WTC signal back to the participant. Wavelet transformation has a limited resolution in time and frequency/period, which leads to boundary effects at places where the wavelet is longer than the available signal time course. The “cone of influence” (COI) marks the area of the wavelet spectrum within which boundary effects are negligible (Torrence & Compo, 1998). To avoid boundary effects at the borders of the window of integration, we must discard coherence values outside the COI. For a period of interest of 14 seconds and a Morlet wavelet with a center frequency of ω_0_= 6, this results in a COI of$\sqrt{2}*\frac{\omega_{0}}{2\pi }*p_{interest}=\sqrt{2}*\frac{6}{2\pi }*14\;s=\;18.9\;s\approx 20\;s$using the COI definition of Matlab’s wavelet toolbox, which follows the definition ofTorrence and Compo (1998). The COI also generally describes the temporal resolution of the wavelet of the corresponding period band. To ensure that no signal from out of our window of integration is leaking in and that all effects measured are only happening within this window, we need to discard about 20 seconds (the greatest length of the COI) at the beginning and end of our window after determining our coherence, meaning discarding almost 40 seconds. This leads to our choice of 50 seconds as the minimal window investigated.Figure 3cdepicts the COI for an exemplary time window and the resulting window after discarding the COI. Since we want to weigh all periods equally, the size of the resulting window is determined by the COI value for the greatest period in the period band of interest.

### 2.5 Short window analysis

A major prerequisite to a neurofeedback study is to ensure that the metric chosen for feedback can be obtained in real time. In conventional fNIRS studies, the entire time course is wavelet transformed at once, which is impossible in a real-time setting where data are only available up to the current time point. To evaluate real-time feasibility, we determined our metric up to the current time point and compared the results for different time windows over which the data were integrated. This was done in simulated data, as well as in existing data where subjects showed higher WTC in the task compared with the rest phases using conventional offline analysis (Kayhan et al., 2022;Pan et al., 2018). We examined whether we could produce a significant contrast as in the offline analysis using short-time window WTC calculations.

As we would do in a real-time neurofeedback experiment, we took the average WTC values of the complete rest phase right before the task phase as the baseline. For the task phase, we took non-overlapping windows of varying lengths and averaged the WTC values within them. We did not use 8 seconds of data at the start of the task phase and between the windows. These offsets account for delays due to the hemodynamic response and ensure the statistical independence of the windows of integration. Note that this means that the placement of the windows differed for each window length and that not all data were used for each window analysis. We compared window lengths of 50, 60, 70, 80, 90, and 100 seconds. Due to our choice of the period band of interest and the corresponding COI, 50 seconds is the lower boundary to the length of the window. We did not investigate window durations longer than 100 seconds since such durations would cause a relatively long delay for neurofeedback training. We compared this window of integration analysis with (1) the analysis of a “complete block,” meaning taking the rest phase and the task phase as one window each and (2) the whole time course of the entire experiment as it would be analyzed for offline analyses. This “offline” analysis is equivalent to the conventional analysis, meaning the complete time course of the whole experiment was first wavelet transformed, and analyses were performed thereafter. The “complete block” analysis can, therefore, be regarded as the full task length window being assessed as in an online setting (calculating the WTC of a cut window), whereas the “offline” analysis is the conventional version (cutting a window of a calculated WTC time course).

### 2.6 Statistical analysis

To assess statistical significance, we first computed the difference between the WTC of each task window (*WTC_task_window_*) and the rest phase (*WTC_rest_*), Δ*WTC= WTC_task_window_*–*WTC_rest_*, and then Fisher-z transformed the differences since differences in WTC are bound between -1 and 1. We then fitted a generalized linear mixed-effects model to the data using MATLAB’s fitglme function. We fitted the formula Δ*WTC ~ 1+(1|dyadID),*with dyadID being the identifier for each dyad, modeling the effect of the dyads as a random intercept. For the model fit, we assumed a normal distribution and used the default maximum pseudo-likelihood as an estimation method.

As a null hypothesis, we assumed that there is no difference between*WTC_task_window_*and*WTC_rest_*. This was rejected if the intercept of the estimated model was significantly different from 0. In the following, the intercept of the estimated model, that is, the fixed effect, is plotted as ΔWTC. For the data re-analyses, we obtained statistical test results for 8 window sizes × 35 channels (music data) and 8 window sizes × 16 channels (RPS data). We corrected the results for multiple testing using a False Discovery Rate (Benjamini & Hochberg, 1995) of q < 0.05.

## 3 Results

### 3.1 Simulations

To assess the theoretical behavior of WTC data for shorter time windows, we simulated artificial dyads using an event-related design.Figure 3depicts the results for our model with a high noise level (SNR = -7.05 dB). It shows a simulated dyad (Fig. 3a), the resulting magnitude scalogram for this particular dyad, that is, the coherence distribution in period and time (Fig. 3b, c), as well as the overall simulation differences of WTC during the task and the rest phase (Fig. 3d). The correspondent results for the model with a moderate noise level (SNR = 0.03 dB) can be found inSupplementary Figure S1.Table 1depicts the results for varying SNR levels from 1.90 to -12.08 dB, averaged over 50 iterations each. Overall, one can state that, as expected, the difference in WTC between the task and rest phases declines with declining SNR levels. The difference between the task and rest phase was not significant only for very low SNR levels of about -12 dB. Importantly, for each of the investigated SNR levels, the difference in WTC stayed at a similar level for each window investigated. The offline results can be replicated for every window investigated. This implies that in our simulations, short windows of WTC are producing a contrast as meaningful as in the established offline condition.

**Table 1.** Simulation results for varying SNR levels.

	50 s	60 s	70 s	80 s	90 s	100 s	Complete	Offline
	***SNR*** ***=*** ***1.90 dB***							
**Δ WTC**	0.446	0.420	0.423	0.415	0.417	0.411	0.412	0.434
**SEM**	0.032	0.031	0.031	0.031	0.031	0.030	0.030	0.029
**p**	3.24E-18	4.19E-16	8.06E-17	1.07E-14	5.56E-15	1.43E-14	3.28E-11	3.54E-12
	***SNR*** ***=*** ***0.03 dB***							
**Δ WTC**	0.396	0.379	0.379	0.376	0.377	0.375	0.374	0.396
**SEM**	0.031	0.030	0.030	0.030	0.030	0.030	0.029	0.029
**p**	1.58E-16	1.06E-13	4.63E-14	5.03E-14	1.78E-14	4.56E-14	7.48E-11	1.83E-11
	***SNR*** ***=*** ***-2.19 dB***							
**Δ WTC**	0.342	0.330	0.327	0.327	0.327	0.326	0.324	0.344
**SEM**	0.030	0.030	0.030	0.030	0.030	0.029	0.029	0.028
**p**	1.58E-13	7.49E-12	2.23E-12	6.55E-11	1.01E-10	8.60E-11	3.31E-09	5.37E-10
	***SNR*** ***=*** ***-7.05 dB***							
**Δ WTC**	0.185	0.179	0.177	0.176	0.176	0.179	0.175	0.185
**SEM**	0.030	0.030	0.029	0.029	0.029	0.029	0.027	0.027
**p**	1.55E-05	4.37E-05	3.66E-05	7.68E-05	6.46E-05	3.70E-05	5.69E-05	2.18E-05
	***SNR*** ***=*** ***-10.15 dB***							
**Δ WTC**	0.099	0.097	0.096	0.096	0.094	0.093	0.091	0.098
**SEM**	0.030	0.030	0.029	0.03	0.03	0.029	0.028	0.028
**p**	0.017	0.019	0.015	0.025	0.028	0.023	0.024	0.016
	***SNR*** ***=*** ***-12.08 dB***							
**Δ WTC**	0.065	0.063	0.062	0.062	0.060	0.060	0.057	0.061
**SEM**	0.030	0.030	0.028	0.029	0.029	0.029	0.027	0.027
**p**	0.100	0.096	0.100	0.121	0.129	0.118	0.125	0.103

The average estimated WTC difference between task and rest does not vary between window sizes and declines with decreasing SNR. SEM is the standard error of the mean. Averages over 50 iterations are displayed. p-values are uncorrected and serve descriptive purposes.

### 3.2 Real data re-analysis

**Fig. 4.**
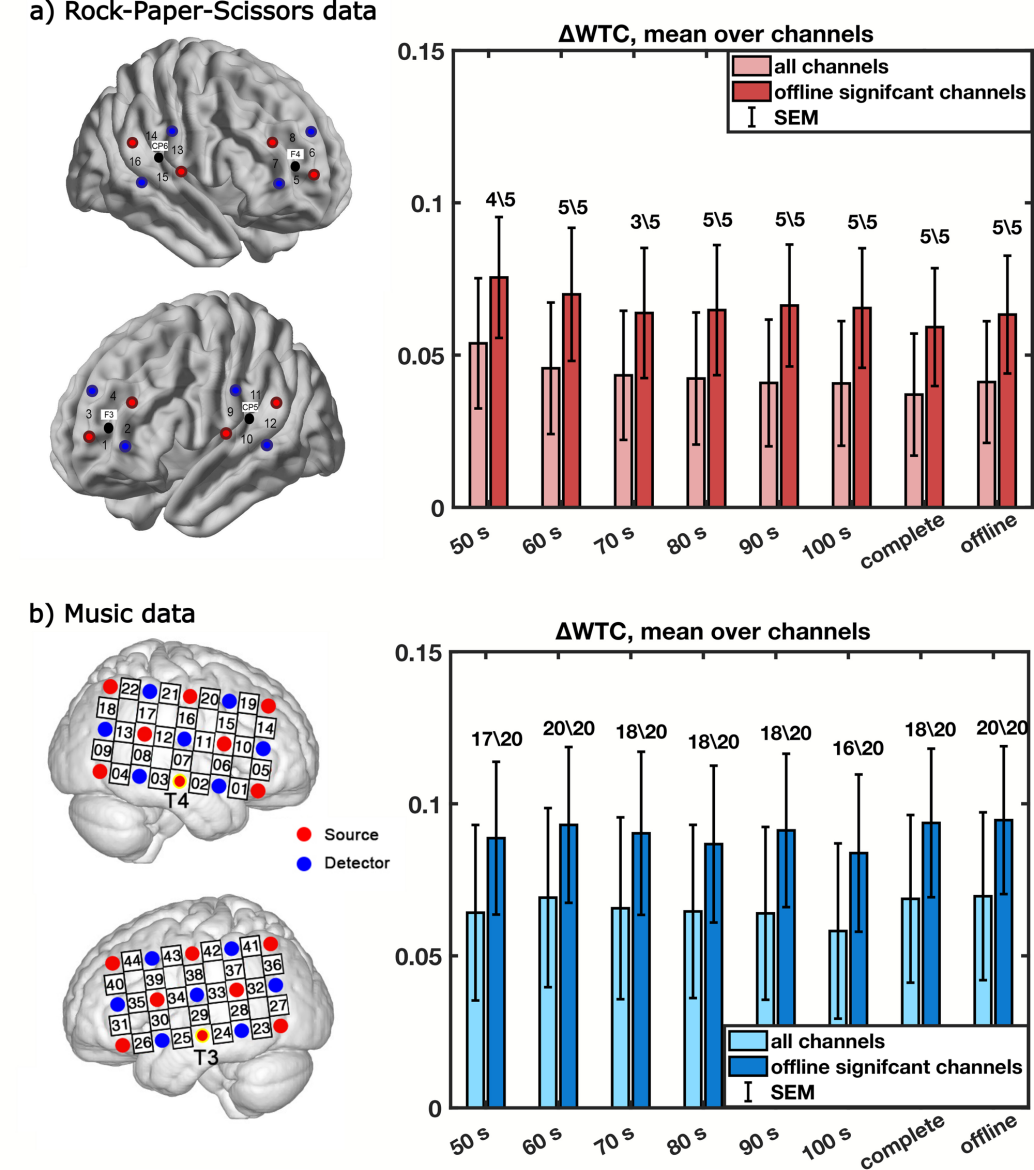
Differences in WTC for different window sizes, reanalyzed for (a) the RPS data set and (b) the music data set. For both data sets, also short windows of WTC depict a significant contrast; ΔWTC does not vary much with window size. For illustration purposes, the mean ΔWTC across all channels is displayed (light red/light blue), as well as the mean across all channels that show a significant contrast both for the specific window size and for the offline analysis (dark red/dark blue). For illustration purposes, we also plotted above the ratio of the number of significant channels for the specific window size out of the total number of offline significant channels. Statistics were performed on a channel and window level, FDR corrected for multiple comparisons at a level of q < 0.05. Single channel and window statistics can be found in the Supplementary Information,Supplementary Figures S2 and S3, andSupplementary Tables S1 and S2.

To complement the simulations, the same analysis was carried out using the actual data of two interaction studies. Both data sets were analyzed conventionally (“offline” condition) and for shorter windows of integration, as would be the case for neurofeedback. AsFigure 4shows, overall, we obtained significant contrasts between the task windows and the rest condition for all investigated window sizes. This holds both for the Rock-Paper-Scissors data set and the music data set. Compared with the simulation results, the variability of the difference in WTC is higher, but one should note that the simulation results depict averages over 50 iterations of 25 dyads, that is, averages over 50 experiments instead of single experiments. In line with the simulation results, we do not observe a systematic effect of window size, neither overall, nor when pre-selecting significant channels. Importantly, statistical testing has been applied to the full data set;Figure 4averages and summarizes the results. The detailed results can be found in the Supplementary Information.Supplementary Figures S2 and S3show the results for each channel and window size separately,Supplementary Tables S1 and S2depict the statistical results for each channel and window size separately. Overall, the real-time windowed analysis of existing data revealed that for many channels, even shorter windows showed a significantly higher synchrony for task than rest, while a pronounced effect of window size was not found.

## 4 Discussion

The aim of this study was to assess the first step of feasibility toward using neurofeedback to train control of interbrain synchrony to improve human interaction. To achieve the goal of neurofeedback, it is essential to investigate whether the current approach of using WTC in fNIRS setups for investigating interbrain synchrony could be technically adapted for real-time analysis. In this manuscript, we examined the feasibility of short time windows of WTC in fNIRS settings by simulating artificial data and by re-analyzing existing data from two hyperscanning experiments.

### 4.1 Feasibility

Our results confirm that short windows of WTC also produce a meaningful contrast between rest and task windows. This shows that the WTC of short data segments is higher in the task condition than in the rest condition. This means that using the WTC signal obtained from shorter time windows, as would be the case in real-time analysis and necessary for neurofeedback, seems technically feasible.

For the re-analysis of existing data, the effect of window length is negligible. This might be because the variability is generally high due to varying noise levels in experimental settings. Also, in our simulations, in the high noise condition, the variability between different iterations is relatively high. Therefore, it can happen that for one iteration of 25 dyads, a shorter window size actually yields a higher contrast than a longer window size. Overall, our results of the real-data re-analysis are consistent with our simulations in high-noise conditions.

These results suggest that shorter window durations likely contain meaningful information for fNIRS hyperfeedback, and longer window durations of up to 100 seconds provide no consistent benefits.

It is perhaps surprising that shorter WTC windows yield as good information as longer windows, given that there are less data available. However, with longer windows, in a real experiment, cognitive processing and engagement likely vary, leading to more fluctuations, which would lead to more variability, potentially counteracting the advantage of averaging over more data. For our simulations, we assumed a constant, event-driven activation. As such, there is no information gain with longer windows. The minimum window size of 50 seconds arises out of the calculations of the COI. Within the COI, a wavelet fully captures the corresponding frequency content. As such, we believe that for our simulations, all relevant information is already fully captured within the smallest window, and variation is only due to noise. It should be noted, however, that this may not be the case anymore for a more complicated data simulation with non-Gaussian noise and varying events.

The high variability of the resulting WTC differences also suggests that either carefully preselecting channels for a subsequent neurofeedback setup or averaging over several channels may be advantageous to reduce the amount of noise being fed back. Overall, our results suggest that these two approaches may be superior to very long integration windows. Longer integration durations can be problematic for neurofeedback, because the number of feedback presentations is reduced, and the temporal contingency between the participants’ actions and the feedback is important for learning self-regulation. Therefore, our results suggest that for our period band chosen, which is a classical range in fNIRS IBS studies, a window size of around 1 minute seems advisable.

We have chosen to operationalize IBS by determining WTC using Morlet wavelets since it is by far the most used metric in fNIRS hyperscanning (Hakim et al., 2023). However, generally, no agreement on measuring IBS has been reached yet (Andrea et al., 2022;Ayrolles et al., 2021;Hakim et al., 2023;Holroyd, 2022), and caution should be taken when generalizing this result to other IBS metrics. Classical correlation measures and WTC do not yield the exact same results in all cases. The Pearson correlation coefficient is dependent on the exact relation of the phases of two signals, whereas WTC does not depend on the exact phase relation as long as the signal is phase locked, meaning that the phase relation does not vary over time. This makes exact comparisons of synchrony results assessed with different metrics difficult. It also implies a slightly different underlying definition of synchrony: with WTC, two signals in one period band can have a constant phase shift up to the corresponding period, and one signal can lead or follow the other. Synchrony will be high as long as this phase difference between the two signals remains constant; see alsoHakim et al. (2023)for a recent review of IBS metrics. Our results, therefore, connect to a substantial body of literature that applied WTC, but cannot be automatically generalized to other synchrony metrics. Also, the choice of the wavelet itself can be optimized: for example,Zhang et al. (2020)used Complex Gaussian wavelets for their analysis to account better for their activation shape. Further work on comparing metrics to obtain an optimal one for different settings of hyperscanning and hyperfeedback is necessary and vital. Also, the relationship between wavelet type, period bands, and head motion should be investigated to determine the most artifact-robust metric. Ideally, this would be done by varying the movement systematically and measuring it with an inertial measurement unit.

The choice of WTC as a metric for IBS also influences the length of the window of integration. Since a signal cannot be localized equally well in frequency/period and time (due to the physical Heisenberg–Gabor limit,Gabor, 1946), our lowest period band of interest determines the temporal resolution. The higher the period, the wider the wavelet in use and the wider the temporal integration of the signal for that period by the signal processing algorithm. This is the wavelet’s COI, which generally describes the “resolution” of a wavelet and, therefore, the temporal resolution of the derived coherence values. This is especially relevant for non-periodic signals, as usually is the case with fNIRS data. Note that the temporal resolution could theoretically be improved by worsening the period resolution. This would mean deviating from the WTC analysis as it is currently used in the field and is, therefore, beyond the scope of this manuscript.

For this study, we can conclude that the WTC metric, as it is currently practiced in the field, can technically be reasonably well assessed using windows of integration that would be suitable for intermittent neurofeedback. However, whether subjects can learn to self-regulate and learn from such a neurofeedback signal also depends on other experimental factors. Such factors would be the feedback modality, the clarity of instructions, the training duration, as well as subject-specific factors such as individual motivation, height of baseline IBS, and effective regulation strategies. How subjects learn to regulate their IBS is an important topic for future research toward which the assessment of technical feasibility is an essential prerequisite.

### 4.2 Usability

The relatively long windows of integration pose a limitation to neurofeedback. Even though it is possible to present a continuous feedback signal based on such a long window of integration, it will be unclear to subjects which action has led to which amount of IBS within the last minute. Given these results, we recommend intermittent neurofeedback instead of continuous neurofeedback. While in continuous neurofeedback, the subjects receive a feedback signal continuously at any time point during their regulation, intermittent neurofeedback only offers a feedback signal at discrete time points delayed or separated from the regulation periods (Hellrung et al., 2018;Lubianiker et al., 2022;Pamplona et al., 2020). Intermittent feedback has been successfully used in the past and in some settings even worked better than continuous feedback (Hellrung et al., 2018;Johnson et al., 2012;Watanabe et al., 2017; but see alsoEmmert et al., 2017). The advantages of intermittent feedback are that more time is available for complex feedback signal computations, and feedback accuracy/validity is improved due to averaging over more data points. Importantly, the participants can focus on self-regulation without the simultaneous processing of a feedback signal, which can cause dual-task interference. In the case of hyperfeedback, continuous feedback would mean that the subjects have to simultaneously attend to another person for social interaction, as well as a feedback modality. In contrast, during an intermittent feedback setup, the participants can fully engage in the interaction and evaluate the feedback subsequently, thus separating the cognitive processes of regulation and appraisal (Johnson et al., 2012;Lubianiker et al., 2022). In the case of a hyperfeedback experiment, this could mean several blocks of 1-minute social interaction with both subjects attending to each other, followed by 8 seconds of feedback with both subjects turning their attention to the feedback modality, for example, a screen showing IBS as an overlap of circles or stylized brains (Chen et al., 2021). Subjects could then take the feedback into account and adapt their regulation strategies during the next 1 minute of interaction.

Such a hyperfeedback setup could potentially be used in any setting that aims to improve interaction quality, such as in couples therapy or joint decision making. It could also be used to train an individual to synchronize with an expert or a pre-recorded time course. This way, an individual could potentially learn another person’s brain state. Additionally, it is important to note that the investigations into short windows of IBS are relevant not only for a potential hyperfeedback setting but also for investigating temporal fluctuations of IBS over the course of one experiment. While in this case, the requirements for preprocessing are less strict since no real-time processing is needed, the limitations around the temporal resolution of the WTC analysis also hold here. Therefore, this study also informs the future use of WTC in investigations of dynamic IBS.

### 4.3 Limitations

One limitation of this study is the preprocessing of the data, that is, the motion correction, filtering, and transformation of raw signal to HbO/HbR values. While an effort was made to choose preprocessing steps that are real-time ready, following the recommendations ofKlein (2024), the data were preprocessed in an offline way, which will need to be adapted. Special attention should be given to the filter. Currently, the low-pass filter inherent to the TDDR routine is based on MATLAB’s “filtfilt” function, which filters the signal backward and forward, and is, therefore, acausal. Since the windows investigated here are relatively long (50 seconds and more), running an acausal filter like MATLAB’s “filtfilt” on the full window length should be possible, and, therefore, our preprocessing should be applicable to intermittent feedback. If, for future usage, the data cannot be used in blocks and causal filters become necessary, special care should be taken because causal filters shift the phase of the signal, which may affect the WTC calculations. Also, in the future, artifact control will be essential and should be further improved to reduce noise. Due to the lack of short-distance channels, we employed a bPCA in this study to correct for extracerebral contributions. Our baseline of 1 minute is relatively short for our current analysis, but this was determined by the experimental design of the original studies and could not be optimized for this secondary analysis. For future experimental studies, longer baselines may be advisable to better capture the full noise structure. Additionally, a closer examination of the noise components identified by the bPCA—particularly regarding the number of components to use—would be beneficial. If it is possible to externally record additional nuisance signals, this approach should be preferred. For instance, short-distance channels should be utilized to measure physiological artifacts online and filter them out (Godet et al., 2023;Klein, 2024;Klein et al., 2022). Inertial measuring units should be used to assess potential motion artifacts (Klein, 2024). Note that there are already different approaches for real-time preprocessing in practice (Klein, 2024;Kohl et al., 2020).

Finally, in our simulations, we have only assumed Gaussian noise. In the future, more specifically tailored noise models could be used to achieve more exact simulations (Gemignani & Gervain, 2021). Additionally, the model assumed for the simulations is a very simple one only modeling each task event onset, likely discarding additionally ongoing activity in the brain areas investigated, which are often discussed in processing social tasks. The simulations should, therefore, be taken as a first proof of concept that short windows of WTC are possible and meaningful, and less as an exact model of the RPS experiment.

### 4.4 Conclusion

Our study provides a fundamental building block toward a real-time fNIRS hyperfeedback setup for IBS. We show that the currently most in use IBS metric, WTC, is calculable and meaningful for short data units. Given the characteristics of the WTC analysis, an intermittent neurofeedback setup seems most suitable. In the future, it will be important to improve the setup and processing pipeline to remove noise as much as possible. Once these technical challenges have been overcome, it will be important to investigate whether and how subjects can make good use of this new type of neurofeedback, to realize the full potential of hyperfeedback.

## Supplementary Material

Supplementary Material

## Data Availability

All code used for this manuscript is openly available. It can be found athttps://github.com/univiemops/hyperfeedback-feasibility. The data re-analyzed for this secondary analysis were obtained fromPan et al. (2018)andKayhan et al. (2022), and the data availability of the original publications applies.
