# Supplementary material for: Investigating short windows of interbrain synchrony: A step toward fNIRS-based hyperfeedback
Source: Imaging Neurosci (Camb). 2025 Jun 17;3:IMAG.a.43. doi: 10.1162/IMAG.a.43 (PMC12319745; doi:10.1162/IMAG.a.43)
Supplement: Supplementary Material [file imag.a.43_supp.pdf]

Supplementary Material

Simulations

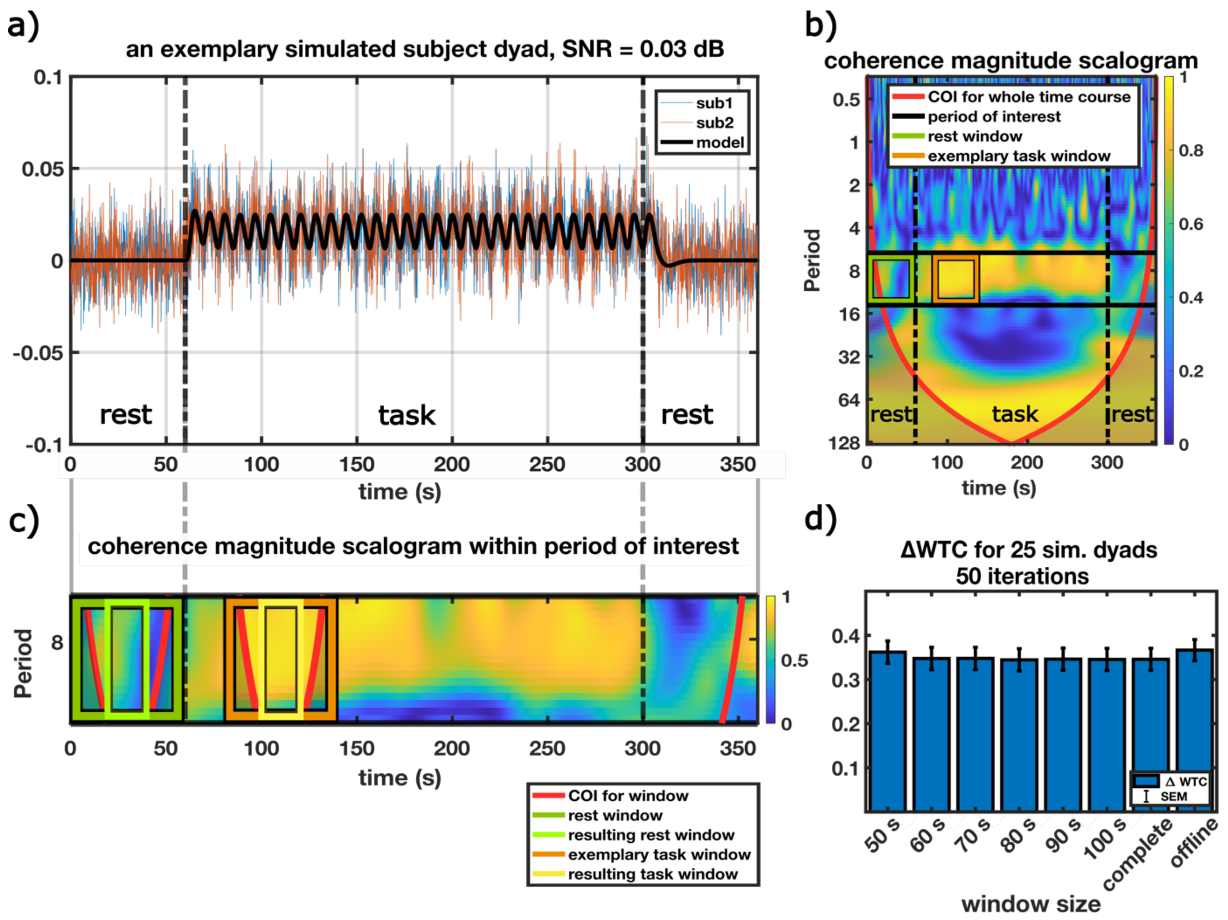

Supplementary Figure 1: Simulated data with a moderate noise level. Figure analog to Figure 3.

Single channel results

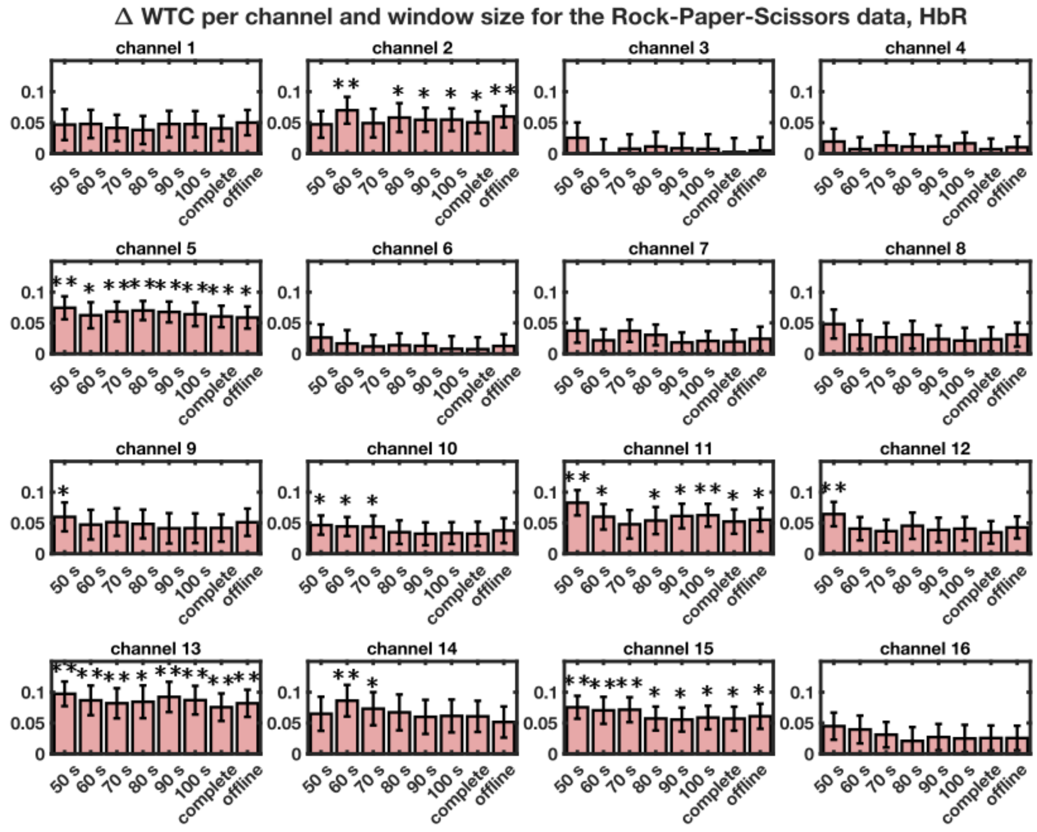

*Supplementary Figure 2: Differences between WTC_{task} and WTC_{rest} for different window sizes and all channels for the Rock-Paper-Scissors data set for HbR. HbR is the chromophore used in the original publication and the manuscript. Results are FDR corrected and marked accordingly. Significance levels: q < 0.05 * and q < 0.01 **.*

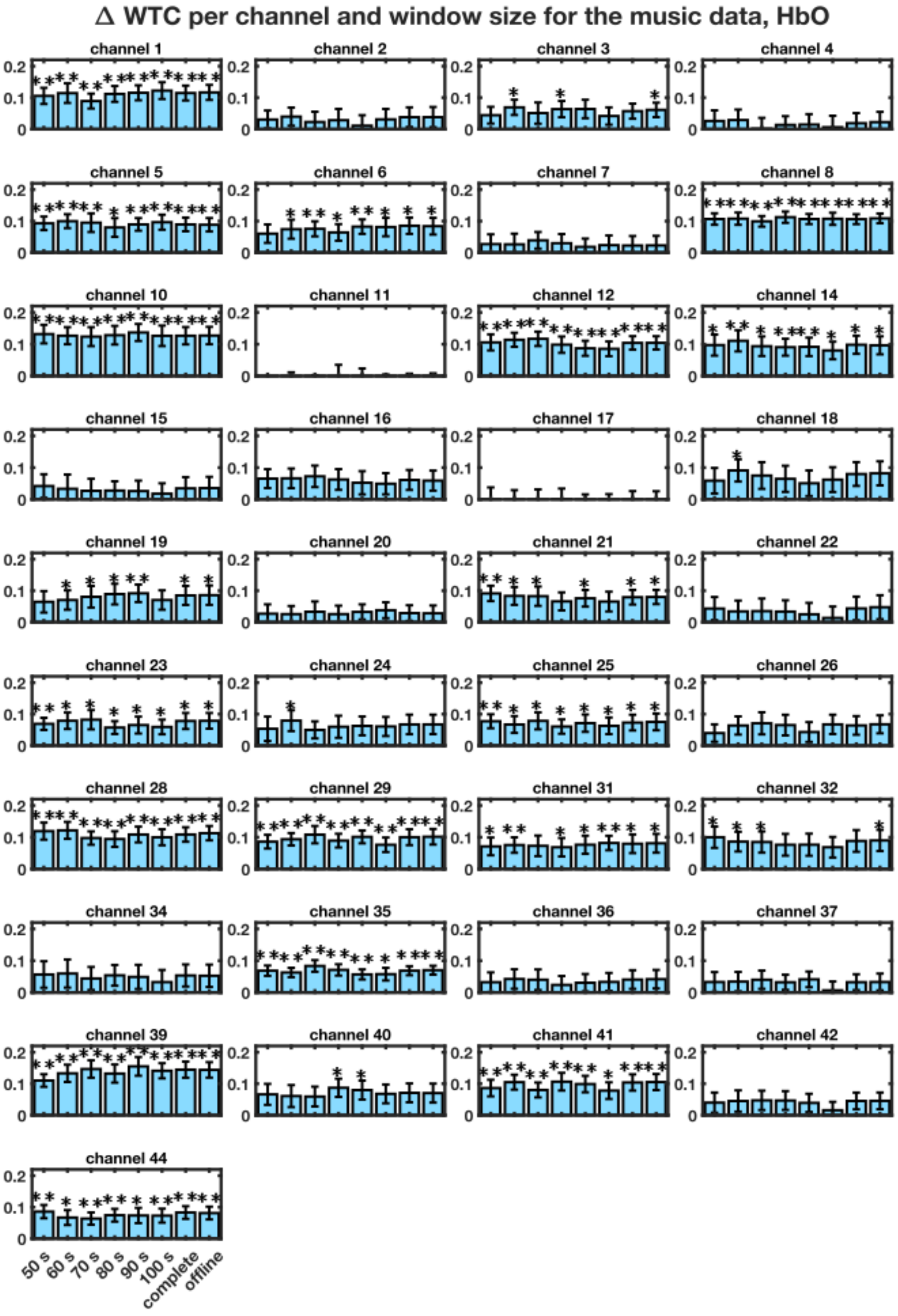

*Supplementary Figure 3: Differences between WTC_{task} and WTC_{rest} for different window sizes and all channels for the music data set for HbO. HbO is the chromophore used in the original publication and in the manuscript. The bar labels of channel 44 apply to all channels. Results are FDR corrected and marked accordingly. Significance levels: $q < 0.05$ * and $q < 0.01$ **.*

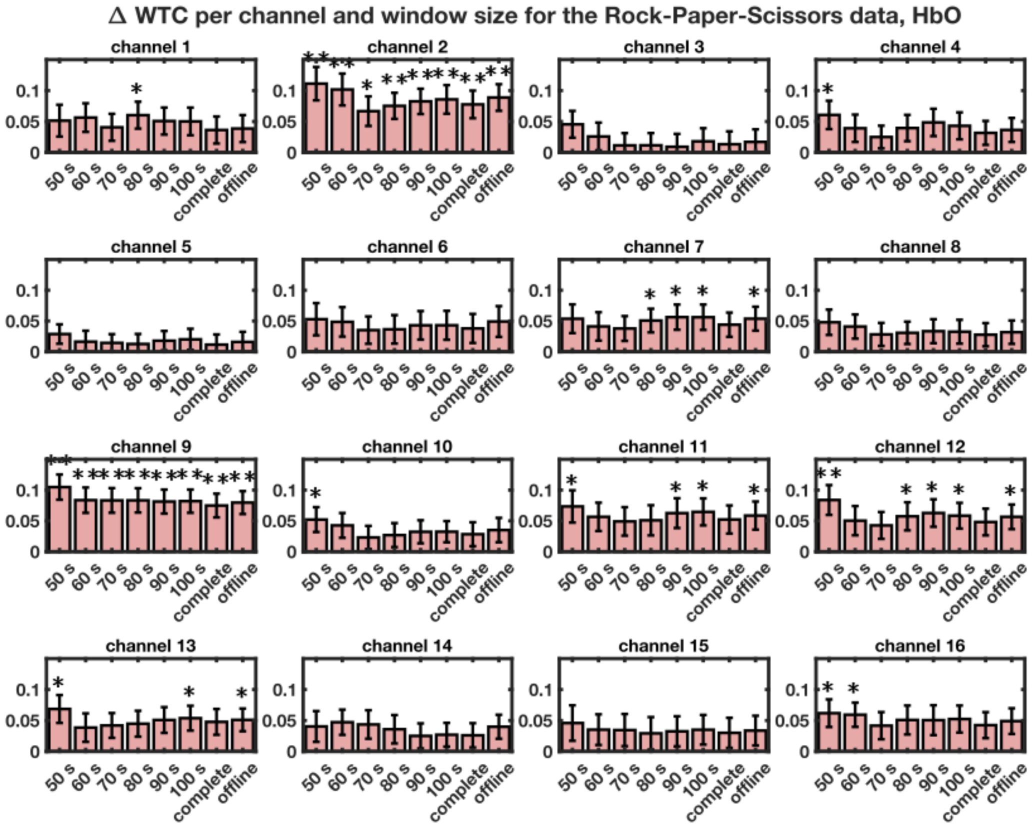

*Supplementary Figure 4: Differences between WTC_{task} and WTC_{rest} for different window sizes and channels for HbO. Results FDR corrected and marked accordingly. Significance levels: q < 0.05 * and q < 0.01 **.*

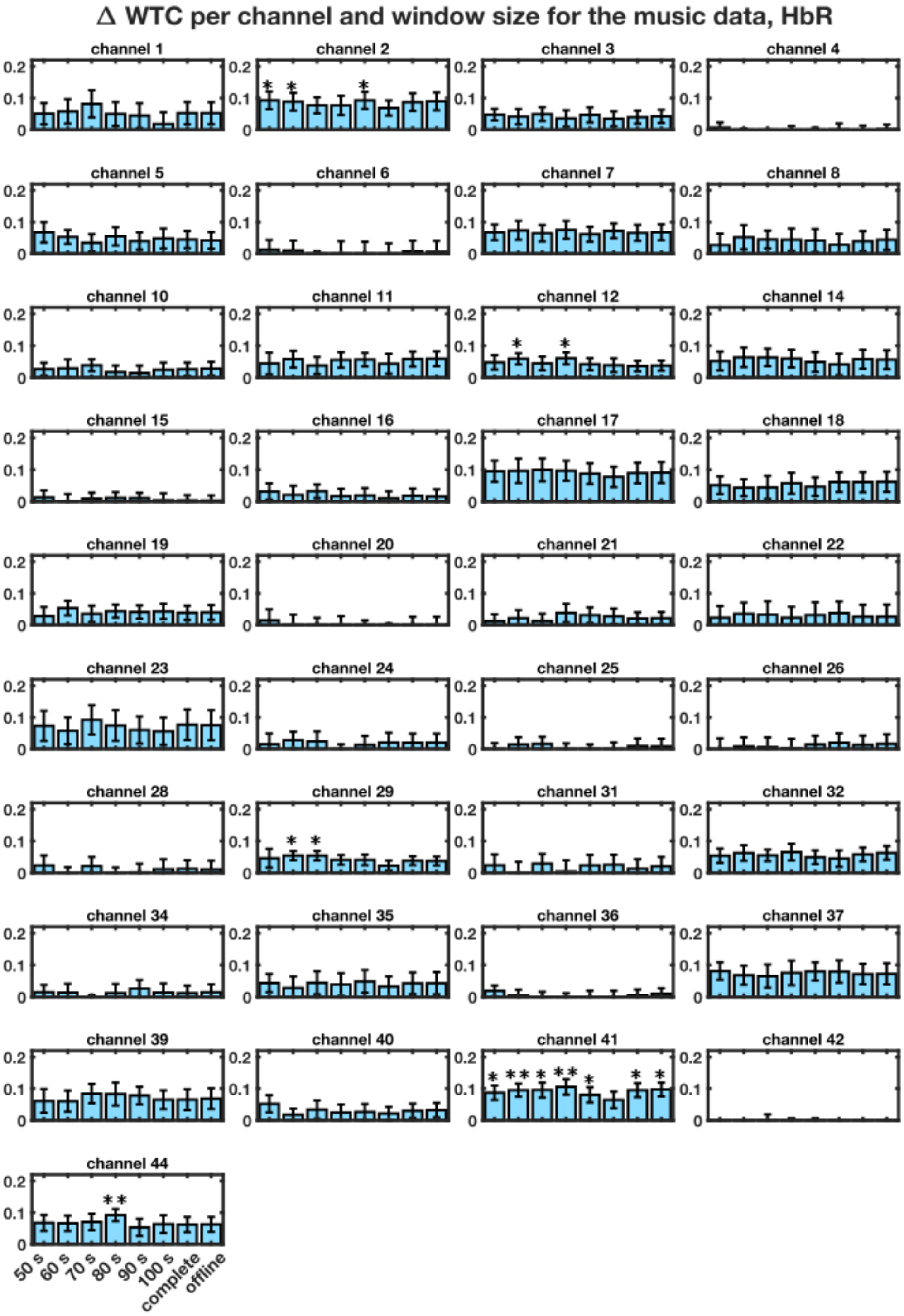

*Supplementary Figure 5: Differences between WTC_{task} and WTC_{rest} for different window sizes and all channels for the music data set for HbR. The bar labels of channel 44 apply to all channels. Results are FDR corrected and marked accordingly. Significance levels: $q < 0.05$ * and $q < 0.01$ **.*

	50 s	60 s	70 s	80 s	90 s	100 s	complete	offline
				channel 1				
Δ WTC	0.105538199	0.114350086	0.08914552	0.11180777	0.115498688	0.122305994	0.114666424	0.116652978
SEM	0.025608334	0.031136641	0.02365189	0.025144874	0.023741508	0.026729362	0.023592189	0.0238962
tStat	4.121244285	3.672524788	3.769065396	4.446543315	4.864842109	4.575716951	4.860355349	4.881653848
DF	83	71	59	47	47	35	11	11
pValues	8.88E-05	0.000461994	0.000381015	5.31E-05	1.33E-05	5.74E-05	0.000502383	0.000485601
Lower	0.054604253	0.052265387	0.041818197	0.061222809	0.067736934	0.068042504	0.062740365	0.064057796
Upper	0.156472144	0.176434785	0.136472842	0.162392732	0.163260441	0.176569483	0.166592483	0.169248161
q<0.05	8.88E-05	0.000461994	0.000381015	5.31E-05	1.33E-05	5.74E-05	0.000502383	0.000485601
q<0.01	8.88E-05	0.000461994	0.000381015	5.31E-05	1.33E-05	5.74E-05	0.000502383	0.000485601
				channel 2				
Δ WTC	0.030534924	0.039705255	0.022612329	0.028391568	0.010967377	0.030987995	0.038020483	0.038095727
SEM	0.028572301	0.028915357	0.032677722	0.035819885	0.03331813	0.033212813	0.03120733	0.032207457
tStat	1.068689715	1.373154605	0.691979978	0.792620296	0.329171454	0.933013271	1.21831901	1.182823188
DF	76	65	54	43	43	32	10	10
pValues	0.288592202	0.174422767	0.491914688	0.432350609	0.743624085	0.357800044	0.2510656	0.264237924
Lower	-0.026371745	-0.0180427	-0.042902558	-0.043846115	-0.056225036	-0.036664291	-0.031513781	-0.03366696
Upper	0.087441594	0.09745321	0.088127216	0.100629251	0.078159791	0.098640282	0.107554747	0.109858414
q<0.05	NaN	NaN	NaN	NaN	NaN	NaN	NaN	NaN
q<0.01	NaN	NaN	NaN	NaN	NaN	NaN	NaN	NaN
				channel 3				
Δ WTC	0.044298817	0.069213315	0.050989189	0.063586824	0.063998323	0.041664403	0.057064541	0.061077863
SEM	0.026452935	0.024205344	0.033654636	0.025879501	0.029344044	0.027451707	0.023935739	0.022984928
tStat	1.674627686	2.859422905	1.515071765	2.457034413	2.180964684	1.517734509	2.384072669	2.65730066
DF	76	65	54	43	43	32	10	10
pValues	0.098117858	0.005699138	0.135586261	0.018119503	0.034702526	0.13889982	0.038344817	0.02401028
Lower	-0.008386769	0.020871905	-0.016484294	0.011395837	0.004820419	-0.014252894	0.003732391	0.009864253
Upper	0.096984403	0.117554726	0.118462671	0.115777811	0.123176228	0.097581701	0.110396692	0.112291474
q<0.05	NaN	0.005699138	NaN	0.018119503	NaN	NaN	NaN	0.02401028
q<0.01	NaN	NaN	NaN	NaN	NaN	NaN	NaN	NaN
				channel 4				
Δ WTC	0.025918226	0.028871037	0.001538597	0.013413408	0.014507151	0.006061246	0.018813229	0.022236588
SEM	0.033638828	0.033260155	0.034190574	0.027731888	0.033035979	0.036419685	0.03213398	0.03266719
tStat	0.770485396	0.868036743	0.045000614	0.483681761	0.439131846	0.166427744	0.585462137	0.680700972
DF	76	65	54	43	43	32	10	10
pValues	0.443400595	0.388567805	0.964272821	0.631065853	0.662764863	0.868867492	0.571211824	0.511513423
Lower	-0.041079307	-0.03755408	-0.067009376	-0.042513274	-0.052116251	-0.068123225	-0.052785741	-0.050550447
Upper	0.092915758	0.095296153	0.07008657	0.069340091	0.081130552	0.080245718	0.090412198	0.095023622
q<0.05	NaN	NaN	NaN	NaN	NaN	NaN	NaN	NaN
q<0.01	NaN	NaN	NaN	NaN	NaN	NaN	NaN	NaN
				channel 5				
Δ WTC	0.092810521	0.099654362	0.094895497	0.07965368	0.089400846	0.096439161	0.089317132	0.088290332
SEM	0.02153124	0.022127181	0.02958935	0.029819499	0.020022445	0.023834278	0.021736839	0.021325588
tStat	4.310505163	4.503708062	3.207082841	2.671194406	4.465031323	4.046237975	4.109021161	4.140112388
DF	83	71	59	47	47	35	11	11
pValues	4.45E-05	2.56E-05	0.002167296	0.010351102	5.00E-05	0.000273053	0.001732661	0.001643728
Lower	0.049985751	0.055534017	0.035687344	0.019664585	0.049120881	0.048053004	0.041474672	0.041353029
Upper	0.135635292	0.143774708	0.15410365	0.139642775	0.129680811	0.144825318	0.137159593	0.135227635
q<0.05	4.45E-05	2.56E-05	0.002167296	0.010351102	5.00E-05	0.000273053	0.001732661	0.001643728
q<0.01	4.45E-05	2.56E-05	0.002167296	NaN	5.00E-05	0.000273053	0.001732661	0.001643728

	50 s	60 s	70 s	80 s	90 s	100 s	complete	offline
				channel 6				
Δ WTC	0.060039156	0.074201557	0.075370808	0.063988585	0.082307453	0.081114602	0.085181872	0.083767762
SEM	0.029313364	0.030254142	0.023830408	0.026285673	0.022960252	0.029253964	0.025930444	0.02656653
tStat	2.048183734	2.452608176	3.162799707	2.434352193	3.584780093	2.772772968	3.285014053	3.153131503
DF	76	65	54	43	43	32	10	10
pValues	0.043996046	0.016875682	0.002564552	0.01914458	0.000855604	0.009190869	0.008218988	0.010278098
Lower	0.001656531	0.013779862	0.027593716	0.010978474	0.036003693	0.021526226	0.027405243	0.024573845
Upper	0.11842178	0.134623251	0.123147899	0.116998695	0.128611214	0.140702977	0.142958502	0.142961679
q<0.05	NaN	0.016875682	0.002564552	0.01914458	0.000855604	0.009190869	0.008218988	0.010278098
q<0.01	NaN	NaN	0.002564552	NaN	0.000855604	NaN	NaN	NaN
				channel 7				
Δ WTC	0.026833377	0.026089502	0.038899067	0.029741592	0.017998165	0.02348867	0.022297793	0.02268561
SEM	0.031072572	0.033887277	0.026440179	0.028946548	0.026270472	0.030638461	0.030240057	0.030391958
tStat	0.863571157	0.769890791	1.471210404	1.027465921	0.685110058	0.766640013	0.737359511	0.746434634
DF	83	71	59	47	47	35	11	11
pValues	0.390312187	0.443918709	0.146548023	0.309460121	0.496639627	0.448435969	0.476338232	0.471057348
Lower	-0.034968719	-0.041479808	-0.01400761	-0.028491352	-0.034851208	-0.038710713	-0.044260122	-0.044206639
Upper	0.088635473	0.093658812	0.091805743	0.087974536	0.070847537	0.085688053	0.088855709	0.089577859
q<0.05	NaN	NaN	NaN	NaN	NaN	NaN	NaN	NaN
q<0.01	NaN	NaN	NaN	NaN	NaN	NaN	NaN	NaN
				channel 8				
Δ WTC	0.10650688	0.107628463	0.099141356	0.11232494	0.106498464	0.107251261	0.106008256	0.108545007
SEM	0.018210766	0.019460628	0.016896542	0.017941305	0.016099289	0.019294143	0.015710845	0.015487504
tStat	5.848566712	5.53057502	5.867552923	6.260689568	6.615103762	5.558747098	6.747457321	7.008553991
DF	76	65	54	43	43	32	10	10
pValues	1.17E-07	6.10E-07	2.78E-07	1.53E-07	4.66E-08	3.91E-06	5.06E-05	3.68E-05
Lower	0.070236996	0.068762905	0.065265828	0.076142851	0.074031155	0.067950378	0.071002312	0.074036698
Upper	0.142776764	0.146494021	0.133016884	0.14850703	0.138965774	0.146552144	0.1410142	0.143053316
q<0.05	1.17E-07	6.10E-07	2.78E-07	1.53E-07	4.66E-08	3.91E-06	5.06E-05	3.68E-05
q<0.01	1.17E-07	6.10E-07	2.78E-07	1.53E-07	4.66E-08	3.91E-06	5.06E-05	3.68E-05
				channel 10				
Δ WTC	0.131848448	0.126716199	0.123843884	0.128371833	0.136904001	0.126460482	0.126620365	0.127225737
SEM	0.029223259	0.026903013	0.029791877	0.02933712	0.026780999	0.032227231	0.027248397	0.027364404
tStat	4.511763991	4.710111814	4.156968195	4.375747678	5.111982648	3.924025724	4.646892218	4.649315071
DF	76	65	54	43	43	32	10	10
pValues	2.30E-05	1.35E-05	0.00011595	7.58E-05	7.01E-06	0.000433365	0.000912355	0.000908991
Lower	0.073645284	0.072987172	0.064114767	0.069207893	0.08289497	0.060815761	0.065907152	0.066254045
Upper	0.190051613	0.180445226	0.183573001	0.187535773	0.190913032	0.192105203	0.187333577	0.188197429
q<0.05	2.30E-05	1.35E-05	0.00011595	7.58E-05	7.01E-06	0.000433365	0.000912355	0.000908991
q<0.01	2.30E-05	1.35E-05	0.00011595	7.58E-05	7.01E-06	0.000433365	0.000912355	0.000908991
				channel 11				
Δ WTC	-0.040436408	-0.026275602	-0.029294251	-0.000496786	-0.011675822	-0.030197885	-0.022690155	-0.021496578
SEM	0.025734791	0.037681778	0.031723028	0.035615907	0.035422001	0.034868721	0.02996837	0.029905587
tStat	-1.571273978	-0.697302594	-0.923438067	-0.013948423	-0.32962061	-0.866045103	-0.757136789	-0.718814784
DF	69	59	49	39	39	29	9	9
pValues	0.120693708	0.488352157	0.360304074	0.988942242	0.74345054	0.393574197	0.468325565	0.490495389
Lower	-0.091775912	-0.101676666	-0.093044062	-0.072536758	-0.083323582	-0.101512427	-0.090483318	-0.089147715
Upper	0.010903096	0.049125462	0.03445556	0.071543186	0.059971939	0.041116657	0.045103007	0.046154559
q<0.05	NaN	NaN	NaN	NaN	NaN	NaN	NaN	NaN
q<0.01	NaN	NaN	NaN	NaN	NaN	NaN	NaN	NaN

Supplementary Table 1: statistical results of the generalized linear mixed-effects model for the music data set for HbO.

	50 s	60 s	70 s	80 s	90 s	100 s	complete	offline
	channel 12							
Δ WTC	0.105733103	0.11394663	0.116957416	0.098534785	0.087131886	0.085454269	0.104125719	0.104264035
SEM	0.025274584	0.022066236	0.022624093	0.025623871	0.023605604	0.023122586	0.021182957	0.020875689
tStat	4.183376609	5.163845365	5.169595865	3.845429324	3.691152541	3.695705543	4.915542198	4.994519353
DF	83	71	59	47	47	35	11	11
pValues	7.09E-05	2.12E-06	2.93E-06	0.000361001	0.000579685	0.000745383	0.000460108	0.000406025
Lower	0.055462972	0.069947805	0.071686711	0.046986206	0.039643536	0.038512924	0.057502345	0.058316952
Upper	0.156003233	0.157945455	0.16222812	0.150083365	0.134620236	0.132395614	0.150749093	0.150211118
q<0.05	7.09E-05	2.12E-06	2.93E-06	0.000361001	0.000579685	0.000745383	0.000460108	0.000406025
q<0.01	7.09E-05	2.12E-06	2.93E-06	0.000361001	0.000579685	0.000745383	0.000460108	0.000406025
	channel 14							
Δ WTC	0.097214822	0.110728188	0.093602863	0.090533119	0.09162624	0.080283606	0.098238903	0.096512173
SEM	0.033225208	0.033395249	0.030853992	0.026615379	0.028982651	0.027742206	0.028417787	0.028281475
tStat	2.925935686	3.31568691	3.033735956	3.401534034	3.161416763	2.89391576	3.456951252	3.41255803
DF	83	71	59	47	47	35	11	11
pValues	0.00442839	0.001442585	0.003588157	0.00137693	0.002748026	0.00650806	0.005361908	0.005798972
Lower	0.031131219	0.044139954	0.031864169	0.036989882	0.033320666	0.023963934	0.035691776	0.034265067
Upper	0.163298424	0.177316423	0.155341558	0.144076356	0.149931814	0.136603277	0.16078603	0.158759279
q<0.05	0.00442839	0.001442585	0.003588157	0.00137693	0.002748026	0.00650806	0.005361908	0.005798972
q<0.01	NaN	0.001442585	NaN	0.00137693	0.002748026	NaN	NaN	NaN
	channel 15							
Δ WTC	0.042013763	0.033324989	0.02697349	0.027732287	0.02622523	0.017896449	0.034789408	0.035718612
SEM	0.037142254	0.044912714	0.037959237	0.029219826	0.032834735	0.032863933	0.035335685	0.035315327
tStat	1.131158146	0.741994548	0.71059094	0.949091442	0.798703868	0.544561988	0.984540346	1.011419534
DF	76	65	54	43	43	32	10	10
pValues	0.261545357	0.460763448	0.480395637	0.347878033	0.428851713	0.589829877	0.348070872	0.335661898
Lower	-0.031961446	-0.056371894	-0.049130198	-0.031195108	-0.039992325	-0.049045192	-0.043943405	-0.04296884
Upper	0.115988972	0.123021872	0.103077178	0.086659681	0.092442785	0.084838089	0.113522221	0.114406064
q<0.05	NaN	NaN	NaN	NaN	NaN	NaN	NaN	NaN
q<0.01	NaN	NaN	NaN	NaN	NaN	NaN	NaN	NaN
	channel 16							
Δ WTC	0.065405851	0.066272413	0.072596007	0.062713738	0.052375521	0.049290226	0.06116674	0.05904566
SEM	0.029708718	0.030961177	0.033841515	0.032610051	0.036530946	0.033371626	0.031378822	0.031587813
tStat	2.201570934	2.140500427	2.145176012	1.923141365	1.433730197	1.477010024	1.949300054	1.86925447
DF	83	71	59	47	47	35	11	11
pValues	0.030471112	0.035750167	0.036066021	0.060532549	0.158268929	0.148615118	0.077209579	0.088425009
Lower	0.006316405	0.004537579	0.004879291	-0.002889223	-0.021115264	-0.018457776	-0.007897582	-0.010478647
Upper	0.124495297	0.128007248	0.140312722	0.128316698	0.125866305	0.117038227	0.130231061	0.128569968
q<0.05	NaN	NaN	NaN	NaN	NaN	NaN	NaN	NaN
q<0.01	NaN	NaN	NaN	NaN	NaN	NaN	NaN	NaN
	channel 17							
Δ WTC	0.000500889	-0.0070564	-0.005009498	0.001313205	-0.022545417	-0.019007974	-0.009206087	-0.009427089
SEM	0.03722834	0.036195994	0.036501725	0.032834813	0.038260011	0.035545207	0.035293685	0.035073464
tStat	0.013454513	-0.194949761	-0.137240041	0.039994284	-0.589268432	-0.534754913	-0.260842319	-0.268781243
DF	83	71	59	47	47	35	11	11
pValues	0.989297464	0.845989068	0.891308335	0.968267093	0.558503196	0.596200526	0.799032858	0.793072333
Lower	-0.073544781	-0.079229162	-0.07804928	-0.064741919	-0.099514632	-0.09116858	-0.086886964	-0.086623264
Upper	0.07454656	0.065116361	0.068030284	0.067368329	0.054423798	0.053152632	0.068474791	0.067769085
q<0.05	NaN	NaN	NaN	NaN	NaN	NaN	NaN	NaN
q<0.01	NaN	NaN	NaN	NaN	NaN	NaN	NaN	NaN

	50 s	60 s	70 s	80 s	90 s	100 s	complete	offline
	channel 18							
Δ WTC	0.058832681	0.091177253	0.075073367	0.064717574	0.050822736	0.062218394	0.079827412	0.08200342
SEM	0.040146928	0.034983748	0.041995018	0.041158032	0.040261267	0.039021621	0.03734804	0.03783152
tStat	1.465434193	2.606274583	1.787673181	1.572416623	1.262323324	1.594459464	2.137392279	2.167595162
DF	69	59	49	39	39	29	9	9
pValues	0.147344688	0.011570462	0.080013853	0.12393259	0.21432899	0.121675278	0.061280565	0.058341526
Lower	-0.021258249	0.021174935	-0.009318781	-0.018532404	-0.030613363	-0.017589783	-0.004659724	-0.003577424
Upper	0.13892361	0.161179571	0.159465514	0.147967553	0.132258834	0.142026571	0.164314548	0.167584265
q<0.05	NaN	0.011570462	NaN	NaN	NaN	NaN	NaN	NaN
q<0.01	NaN	NaN	NaN	NaN	NaN	NaN	NaN	NaN
	channel 19							
Δ WTC	0.06460334	0.070424418	0.080691239	0.089310187	0.091722531	0.070388432	0.085013029	0.085792002
SEM	0.034075831	0.030760931	0.034233612	0.032875696	0.027918483	0.0308229	0.030187835	0.030752542
tStat	1.895869846	2.289411104	2.357076382	2.716602154	3.285369458	2.283640786	2.816135355	2.789753161
DF	83	71	59	47	47	35	11	11
pValues	0.061457282	0.025031797	0.021757168	0.009202047	0.001929585	0.028574068	0.016783299	0.017594303
Lower	-0.003172117	0.009088863	0.012189939	0.023172817	0.035557788	0.007814618	0.018570052	0.018106113
Upper	0.132378796	0.131759973	0.149192539	0.155447556	0.147887274	0.132962246	0.151456006	0.153477891
q<0.05	NaN	0.025031797	0.021757168	0.009202047	0.001929585	NaN	0.016783299	0.017594303
q<0.01	NaN	NaN	NaN	NaN	0.001929585	NaN	NaN	NaN
	channel 20							
Δ WTC	0.026897946	0.025544829	0.033133412	0.025580305	0.032613526	0.037918036	0.028882373	0.028171525
SEM	0.029825459	0.025809358	0.032690689	0.026956352	0.024169624	0.025191121	0.024963292	0.0249435
tStat	0.901845172	0.989750632	1.013542792	0.948952791	1.34936005	1.505214323	1.15699374	1.129413512
DF	83	71	59	47	47	35	11	11
pValues	0.36974876	0.325657129	0.314939511	0.34749876	0.183685863	0.1412411	0.271782521	0.282751055
Lower	-0.032423692	-0.02591757	-0.032280505	-0.02864888	-0.016009487	-0.013222658	-0.026061463	-0.026728747
Upper	0.086219585	0.077007228	0.098547329	0.07980949	0.081236538	0.089058731	0.083826208	0.083071798
q<0.05	NaN	NaN	NaN	NaN	NaN	NaN	NaN	NaN
q<0.01	NaN	NaN	NaN	NaN	NaN	NaN	NaN	NaN
	channel 21							
Δ WTC	0.091376005	0.083336107	0.082558714	0.0660793	0.075843495	0.06547477	0.079324519	0.080044166
SEM	0.02438885	0.028316612	0.030658147	0.028606181	0.026395385	0.031340955	0.023238413	0.022576462
tStat	3.746630367	2.943011238	2.692880098	2.309965792	2.87336196	2.089112139	3.413508473	3.54546991
DF	76	65	54	43	43	32	10	10
pValues	0.000346995	0.004502322	0.009415446	0.025755417	0.006286259	0.044735281	0.006619171	0.005308151
Lower	0.042801401	0.026783928	0.02109283	0.008389438	0.022612128	0.001635333	0.027546109	0.029740674
Upper	0.139950609	0.139888287	0.144024598	0.123769163	0.129074863	0.129314207	0.131102929	0.130347658
q<0.05	0.000346995	0.004502322	0.009415446	NaN	0.006286259	NaN	0.006619171	0.005308151
q<0.01	0.000346995	NaN	NaN	NaN	NaN	NaN	NaN	NaN
	channel 22							
Δ WTC	0.043225392	0.03427612	0.035060641	0.033623087	0.02505097	0.013456409	0.04366132	0.047502833
SEM	0.03675458	0.034396181	0.040042058	0.035965735	0.03629808	0.03628051	0.037508979	0.038106912
tStat	1.176054593	0.996509448	0.875595367	0.934864435	0.690145871	0.370899104	1.164023159	1.246567369
DF	76	65	54	43	43	32	10	10
pValues	0.243243851	0.322698017	0.385128254	0.355078733	0.493810789	0.713156856	0.271434508	0.240965172
Lower	-0.029977698	-0.034417798	-0.045218853	-0.038908731	-0.048151085	-0.060444572	-0.039913893	-0.037404658
Upper	0.116428482	0.102970037	0.115340134	0.106154904	0.098253026	0.087357389	0.127236534	0.132410324
q<0.05	NaN	NaN	NaN	NaN	NaN	NaN	NaN	NaN
q<0.01	NaN	NaN	NaN	NaN	NaN	NaN	NaN	NaN

Continuation Supplementary Table 1

	50 s	60 s	70 s	80 s	90 s	100 s	complete	offline
	channel 23							
Δ WTC	0.069061975	0.078935771	0.082347019	0.057302096	0.065264307	0.059240045	0.078230479	0.078754613
SEM	0.01929098	0.026113956	0.030694399	0.020382695	0.026747514	0.02328844	0.025503936	0.024976245
tStat	3.580013874	3.022742761	2.682802746	2.811311046	2.440013904	2.543753264	3.06738841	3.153180707
DF	69	59	49	39	39	29	9	9
pValues	0.000634467	0.003702696	0.009926183	0.007680827	0.019334296	0.016556626	0.013410481	0.011677627
Lower	0.030577524	0.026681866	0.020664314	0.016074204	0.011162353	0.011609837	0.020536566	0.022254422
Upper	0.107546427	0.131189677	0.144029724	0.098529988	0.119366261	0.106870253	0.135924391	0.135254804
q<0.05	0.000634467	0.003702696	0.009926183	0.007680827	0.019334296	0.016556626	0.013410481	0.011677627
q<0.01	0.000634467	NaN	NaN	NaN	NaN	NaN	NaN	NaN
	channel 24							
Δ WTC	0.053496926	0.079358352	0.049872061	0.059825882	0.062589033	0.06072538	0.067267303	0.067353777
SEM	0.03865075	0.033325242	0.027116932	0.035127945	0.029900846	0.030296735	0.031111962	0.030947606
tStat	1.384110926	2.381328649	1.839148339	1.703085173	2.093219471	2.004353951	2.162104181	2.176380861
DF	83	71	59	47	47	35	11	11
pValues	0.170034981	0.019934645	0.070926953	0.095157313	0.041752627	0.05281783	0.053507915	0.052193459
Lower	-0.023377862	0.012909708	-0.004388795	-0.010842428	0.00243629	-0.000780261	-0.001209664	-0.000761444
Upper	0.130371714	0.145806997	0.104132916	0.130494192	0.122741777	0.122231021	0.135744269	0.135468998
q<0.05	NaN	0.019934645	NaN	NaN	NaN	NaN	NaN	NaN
q<0.01	NaN	NaN	NaN	NaN	NaN	NaN	NaN	NaN
	channel 25							
Δ WTC	0.077405471	0.067056163	0.078558559	0.060465512	0.071329493	0.062814567	0.073111749	0.075208061
SEM	0.022204057	0.026691621	0.026795837	0.022720073	0.027594218	0.026408163	0.024969199	0.026588281
tStat	3.486095767	2.512255196	2.931744921	2.661325582	2.584943445	2.378604212	2.928077414	2.828616859
DF	69	59	49	39	39	29	9	9
pValues	0.000856778	0.014750918	0.005110368	0.011243707	0.013594972	0.024181917	0.016809488	0.019766983
Lower	0.033109588	0.013646353	0.024710309	0.014509826	0.015514919	0.00880381	0.016627496	0.01506119
Upper	0.121701353	0.120465972	0.13240681	0.106421197	0.127144067	0.116825325	0.129596002	0.135354932
q<0.05	0.000856778	0.014750918	0.005110368	0.011243707	0.013594972	0.024181917	0.016809488	0.019766983
q<0.01	0.000856778	NaN	NaN	NaN	NaN	NaN	NaN	NaN
	channel 26							
Δ WTC	0.039807245	0.063867182	0.070775008	0.065169217	0.043122151	0.067432977	0.064095564	0.067657515
SEM	0.027707281	0.02884034	0.035110688	0.032746869	0.031542126	0.030690783	0.029583519	0.028513384
tStat	1.436707022	2.2145086	2.015768168	1.990090023	1.367128836	2.19717357	2.166597006	2.372833572
DF	83	71	59	47	47	35	11	11
pValues	0.154560116	0.030004937	0.048385749	0.052415668	0.178088345	0.034718726	0.053090902	0.036966338
Lower	-0.015301422	0.006361176	0.000518683	-0.000708986	-0.020332423	0.005127375	-0.001017323	0.00489998
Upper	0.094915913	0.121373187	0.141031332	0.13104742	0.106576724	0.129738579	0.129208451	0.13041505
q<0.05	NaN	NaN	NaN	NaN	NaN	NaN	NaN	NaN
q<0.01	NaN	NaN	NaN	NaN	NaN	NaN	NaN	NaN
	channel 28							
Δ WTC	0.119281367	0.121696618	0.097468916	0.094357421	0.108607805	0.09997636	0.108688628	0.112826272
SEM	0.02682198	0.026698014	0.021041502	0.024645676	0.024761516	0.025182281	0.022720985	0.022594226
tStat	4.447149959	4.558264866	4.632222388	3.828558796	4.386153296	3.970107399	4.783623028	4.993588742
DF	83	71	59	47	47	35	11	11
pValues	2.68E-05	2.10E-05	2.04E-05	0.000380343	6.47E-05	0.000340373	0.000568096	0.000406621
Lower	0.065933528	0.068462291	0.055364968	0.044776715	0.05879406	0.048853612	0.058680077	0.063096716
Upper	0.172629206	0.174930946	0.139572864	0.143938126	0.15842155	0.151099108	0.15869718	0.162555827
q<0.05	2.68E-05	2.10E-05	2.04E-05	0.000380343	6.47E-05	0.000340373	0.000568096	0.000406621
q<0.01	2.68E-05	2.10E-05	2.04E-05	0.000380343	6.47E-05	0.000340373	0.000568096	0.000406621

	50 s	60 s	70 s	80 s	90 s	100 s	complete	offline
	channel 29							
Δ WTC	0.086051847	0.093672679	0.108211107	0.089321931	0.101160757	0.076314353	0.100213642	0.101557681
SEM	0.021642338	0.019450647	0.027296304	0.021652394	0.02022478	0.022849105	0.025030573	0.024572399
tStat	3.976088272	4.815915957	3.964313469	4.125268217	5.00182235	3.339927443	4.003649534	4.132998254
DF	76	65	54	43	43	32	10	10
pValues	0.000158386	9.13E-06	0.000217967	0.000166403	1.01E-05	0.002139939	0.002503554	0.002034432
Lower	0.042947395	0.054827055	0.053485312	0.045655718	0.060373601	0.029772249	0.04444205	0.046806965
Upper	0.129156299	0.132518303	0.162936902	0.132988144	0.141947914	0.122856457	0.155985234	0.156308398
q<0.05	0.000158386	9.13E-06	0.000217967	0.000166403	1.01E-05	0.002139939	0.002503554	0.002034432
q<0.01	0.000158386	9.13E-06	0.000217967	0.000166403	1.01E-05	0.002139939	0.002503554	0.002034432
	channel 31							
Δ WTC	0.071155117	0.07546709	0.07295173	0.068506484	0.07646442	0.08192951	0.079537543	0.081031424
SEM	0.027910347	0.024308295	0.032428612	0.02908532	0.028485523	0.022661412	0.029283196	0.02957233
tStat	2.549417169	3.104581807	2.249610026	2.35536295	2.684325645	3.615375326	2.716149689	2.740109576
DF	83	71	59	47	47	35	11	11
pValues	0.012629711	0.002736643	0.028218368	0.022729935	0.010005949	0.000934414	0.020069092	0.019227678
Lower	0.01564256	0.026997727	0.008062228	0.009994368	0.019158939	0.035924398	0.015085664	0.015943165
Upper	0.126667675	0.123936454	0.137841233	0.1270186	0.1337699	0.127934622	0.143989423	0.146119683
q<0.05	0.012629711	0.002736643	NaN	0.022729935	0.010005949	0.000934414	0.020069092	0.019227678
q<0.01	NaN	0.002736643	NaN	NaN	NaN	0.000934414	NaN	NaN
	channel 32							
Δ WTC	0.100080555	0.086191489	0.085062588	0.07635045	0.076552955	0.068560373	0.088410518	0.089980785
SEM	0.033598608	0.030978243	0.033495263	0.034299282	0.034787246	0.03238921	0.03412522	0.033430865
tStat	2.978711389	2.782323307	2.539540856	2.226007234	2.200604057	2.116765871	2.590767735	2.691548247
DF	76	65	54	43	43	32	10	10
pValues	0.003883809	0.007056866	0.014011562	0.031306296	0.033183549	0.04214858	0.026914484	0.02263945
Lower	0.033163129	0.024323664	0.01790863	0.007179355	0.006397787	0.002585712	0.01237479	0.015492177
Upper	0.166997981	0.148059313	0.152216546	0.145521545	0.146708123	0.134535034	0.164446246	0.164469394
q<0.05	0.003883809	0.007056866	0.014011562	NaN	NaN	NaN	NaN	0.02263945
q<0.01	NaN	NaN	NaN	NaN	NaN	NaN	NaN	NaN
	channel 34							
Δ WTC	0.056503359	0.059496363	0.043970459	0.053849065	0.048881325	0.032563554	0.053461292	0.052538395
SEM	0.041741508	0.044323604	0.036249365	0.03265584	0.038020705	0.038152771	0.034754231	0.035112776
tStat	1.353649221	1.342317815	1.212999419	1.648987265	1.285650154	0.8535043	1.538267185	1.496275745
DF	69	59	49	39	39	29	9	9
pValues	0.180265964	0.184635429	0.230945486	0.107183665	0.206146942	0.400374835	0.158364515	0.168800098
Lower	-0.02676867	-0.029194963	-0.028875368	-0.012203607	-0.028022809	-0.045467624	-0.025158239	-0.026892223
Upper	0.139775389	0.148187689	0.116816286	0.119901737	0.12578546	0.110594732	0.132080824	0.131969013
q<0.05	NaN	NaN	NaN	NaN	NaN	NaN	NaN	NaN
q<0.01	NaN	NaN	NaN	NaN	NaN	NaN	NaN	NaN
	channel 35							
Δ WTC	0.068492537	0.062782426	0.083398763	0.070991741	0.056782705	0.057481273	0.068412297	0.070059512
SEM	0.016284845	0.014725636	0.018308184	0.017790147	0.015855461	0.019931739	0.014190155	0.013949064
tStat	4.205906638	4.263478146	4.555272242	3.990509031	3.581271178	2.883906545	4.821110004	5.022524215
DF	76	65	54	43	43	32	10	10
pValues	7.04E-05	6.65E-05	3.03E-05	0.000252332	0.000864485	0.006969751	0.000701059	0.000519806
Lower	0.036058458	0.033373301	0.046693064	0.035114491	0.024807121	0.016881649	0.036794662	0.038979061
Upper	0.100926617	0.092191551	0.120104463	0.106868991	0.088758289	0.098080896	0.100029932	0.101139964
q<0.05	7.04E-05	6.65E-05	3.03E-05	0.000252332	0.000864485	0.006969751	0.000701059	0.000519806
q<0.01	7.04E-05	6.65E-05	3.03E-05	0.000252332	0.000864485	NaN	0.000701059	0.000519806

Continuation Supplementary Table 1

	50 s	60 s	70 s	80 s	90 s	100 s	complete	offline
	channel 36							
Δ WTC	0.032233338	0.042298011	0.040041013	0.023647464	0.030503344	0.033124395	0.040533568	0.041737135
SEM	0.030903687	0.030688316	0.032388585	0.02808248	0.027504283	0.028256369	0.029337951	0.029098799
tStat	1.043025669	1.378309965	1.236269276	0.842071792	1.109039781	1.172280674	1.381608709	1.43432501
DF	83	71	59	47	47	35	11	11
pValues	0.299964886	0.172435576	0.2212576	0.404012327	0.273055723	0.249002125	0.194507947	0.179291506
Lower	-0.029232851	-0.018892752	-0.024768397	-0.032847199	-0.024828136	-0.024239083	-0.024038826	-0.02230889
Upper	0.093699528	0.103488775	0.104850422	0.080142128	0.085834823	0.090487873	0.105105963	0.10578316
q<0.05	NaN	NaN	NaN	NaN	NaN	NaN	NaN	NaN
q<0.01	NaN	NaN	NaN	NaN	NaN	NaN	NaN	NaN
	channel 37							
Δ WTC	0.032812059	0.034091049	0.04001204	0.032031328	0.041267752	0.00655612	0.032262954	0.032860992
SEM	0.031331784	0.030459371	0.028819232	0.023768284	0.024615019	0.027961793	0.024977198	0.026558134
tStat	1.047245179	1.11923025	1.388379823	1.347650013	1.676527343	0.23446709	1.291696267	1.237323101
DF	69	59	49	39	39	29	9	9
pValues	0.298641153	0.267577886	0.171301222	0.185548039	0.101634381	0.816269928	0.228645988	0.247276188
Lower	-0.029693139	-0.026858011	-0.017902375	-0.016044564	-0.008520823	-0.050632167	-0.024239394	-0.02721768
Upper	0.095317258	0.095040109	0.097926455	0.080107221	0.091056327	0.063744407	0.088765301	0.092939664
q<0.05	NaN	NaN	NaN	NaN	NaN	NaN	NaN	NaN
q<0.01	NaN	NaN	NaN	NaN	NaN	NaN	NaN	NaN
	channel 39							
Row	windowsize 50 s	windowsize 60 s	windowsize 70 s	windowsize 80 s	windowsize 90 s	windowsize 100	complete block	offline
Δ WTC	0.110287443	0.133089039	0.146766273	0.132383481	0.15519066	0.141221289	0.144800099	0.144083316
SEM	0.020009336	0.027458031	0.027577204	0.028860744	0.029741205	0.024073713	0.025580685	0.024564629
tStat	5.511799342	4.84699856	5.322014224	4.586974016	5.218035353	5.866203013	5.660524575	5.865478971
DF	69	59	49	39	39	29	9	9
pValues	5.74E-07	9.48E-06	2.54E-06	4.57E-05	6.28E-06	2.30E-06	0.000309429	0.000239105
Lower	0.070369911	0.078145645	0.091347807	0.074007117	0.095033394	0.091985017	0.086932568	0.088514264
Upper	0.150204976	0.188032433	0.20218474	0.190759845	0.215347925	0.19045756	0.20266763	0.199652369
q<0.05	5.74E-07	9.48E-06	2.54E-06	4.57E-05	6.28E-06	2.30E-06	0.000309429	0.000239105
q<0.01	5.74E-07	9.48E-06	2.54E-06	4.57E-05	6.28E-06	2.30E-06	0.000309429	0.000239105
	channel 40							
Δ WTC	0.066236835	0.060885263	0.059314622	0.087217663	0.079957858	0.067874348	0.071036906	0.070330056
SEM	0.033277212	0.035019485	0.031449701	0.028867096	0.030091553	0.029831969	0.029914918	0.03006868
tStat	1.990456284	1.738611025	1.886015418	3.021352123	2.657152896	2.27522187	2.374631503	2.338980538
DF	83	71	59	47	47	35	11	11
pValues	0.049831813	0.086437905	0.064218216	0.004063063	0.010732252	0.029126235	0.036849081	0.039242785
Lower	4.98E-05	-0.008941607	-0.003616085	0.029144556	0.019421461	0.007312231	0.005194616	0.004149339
Upper	0.132423871	0.130712133	0.122245329	0.14529077	0.140494255	0.128436464	0.136879196	0.136510774
q<0.05	NaN	NaN	NaN	0.004063063	0.010732252	NaN	NaN	NaN
q<0.01	NaN	NaN	NaN	NaN	NaN	NaN	NaN	NaN
	channel 41							
Δ WTC	0.086284662	0.104996143	0.080225033	0.10658308	0.099020274	0.078141355	0.104022925	0.105379937
SEM	0.026298796	0.023669666	0.0236447	0.028114364	0.026179402	0.026448602	0.025892223	0.025561817
tStat	3.280935832	4.435894604	3.392939346	3.791054334	3.782373457	2.954460656	4.017535472	4.122552637
DF	83	71	59	47	47	35	11	11
pValues	0.001514114	3.28E-05	0.001240455	0.000426987	0.000438544	0.005569309	0.002024616	0.001693354
Lower	0.033977415	0.05780017	0.032912098	0.050024276	0.046354111	0.024447838	0.047034526	0.049118757
Upper	0.13859191	0.152192116	0.127537969	0.163141885	0.151686437	0.131834873	0.161011324	0.161641118
q<0.05	0.001514114	3.28E-05	0.001240455	0.000426987	0.000438544	0.005569309	0.002024616	0.001693354
q<0.01	0.001514114	3.28E-05	0.001240455	0.000426987	0.000438544	NaN	0.002024616	0.001693354

	50 s	60 s	70 s	80 s	90 s	100 s	complete	offline
	channel 42							
Row	windowsize 50 s	windowsize 60 s	windowsize 70 s	windowsize 80 s	windowsize 90 s	windowsize 100	complete block	offline
Δ WTC	0.039976092	0.045189515	0.047244656	0.046738319	0.039203213	0.015866058	0.045369528	0.045694613
SEM	0.032059523	0.033773209	0.03073416	0.029780581	0.028861298	0.026567171	0.026498906	0.026511586
tStat	1.246933474	1.338028489	1.537203423	1.569422644	1.358331595	0.597205382	1.712128378	1.723571465
DF	76	65	54	43	43	32	10	10
pValues	0.216251098	0.185549452	0.130082847	0.123879815	0.181442677	0.554572828	0.117653593	0.115505872
Lower	-0.023875981	-0.02226024	-0.014373625	-0.013319947	-0.019001141	-0.038249499	-0.013673713	-0.013376882
Upper	0.103828165	0.11263927	0.108862937	0.106796585	0.097407567	0.069981615	0.10441277	0.104766107
q<0.05	NaN	NaN	NaN	NaN	NaN	NaN	NaN	NaN
q<0.01	NaN	NaN	NaN	NaN	NaN	NaN	NaN	NaN
	channel 44							
Row	windowsize 50 s	windowsize 60 s	windowsize 70 s	windowsize 80 s	windowsize 90 s	windowsize 100	complete block	offline
Δ WTC	0.085873786	0.067032498	0.063694281	0.074393578	0.073540249	0.073095815	0.083019444	0.08135035
SEM	0.021119569	0.023689293	0.019451664	0.020210226	0.024249432	0.022149967	0.020100108	0.020297479
tStat	4.066076665	2.829653781	3.274490059	3.680986961	3.03265866	3.300041641	4.130298386	4.007904093
DF	83	71	59	47	47	35	11	11
pValues	0.000108217	0.006054531	0.001773827	0.000597882	0.003938164	0.002229535	0.001671274	0.0020582
Lower	0.043867813	0.019797389	0.024771591	0.033735848	0.024756685	0.02812899	0.038779404	0.036675899
Upper	0.127879759	0.114267608	0.102616971	0.115051308	0.122323812	0.118062639	0.127259483	0.1260248
q<0.05	0.000108217	0.006054531	0.001773827	0.000597882	0.003938164	0.002229535	0.001671274	0.0020582
q<0.01	0.000108217	NaN	0.001773827	0.000597882	NaN	0.002229535	0.001671274	0.0020582

Continuation Supplementary Table 1

	50 s	60 s	70 s	80 s	90 s	100 s	complete	offline
				channel 1				
Δ WTC	0.046835881	0.04786937	0.04154066	0.038161066	0.047790469	0.047650001	0.040744876	0.050071411
SEM	0.024895294	0.02275541	0.021136872	0.022659086	0.021284828	0.021075164	0.020164442	0.020376928
tStat	1.881314662	2.103647875	1.965317282	1.684139674	2.245283347	2.260955159	2.020630024	2.457260094
DF	191	143	143	95	95	95	47	47
pValues	0.061450525	0.037159909	0.051315176	0.095437714	0.027068854	0.026043873	0.049036393	0.017745681
Lower	-0.00226914	0.002888928	-0.00024043	-0.006822908	0.005534744	0.00581051	0.000179252	0.00907832
Upper	0.095940902	0.092849812	0.08332175	0.083145039	0.090046194	0.089489491	0.081310501	0.091064502
q<0.05	NaN	NaN	NaN	NaN	NaN	NaN	NaN	NaN
q<0.01	NaN	NaN	NaN	NaN	NaN	NaN	NaN	NaN
				channel 2				
Δ WTC	0.047258085	0.069988529	0.049234658	0.058187743	0.054589769	0.054828066	0.050563801	0.059731913
SEM	0.021584598	0.021627837	0.023064895	0.023246498	0.019315757	0.018137555	0.017752495	0.017434115
tStat	2.189435463	3.236039204	2.134614442	2.503075675	2.826178167	3.022902846	2.848264394	3.426151129
DF	183	137	137	91	91	91	45	45
pValues	0.029830462	0.001519777	0.034572643	0.014095837	0.005790575	0.003252033	0.00660424	0.001317694
Lower	0.004671415	0.027220969	0.003625415	0.012011431	0.016221392	0.018800042	0.014808439	0.024617803
Upper	0.089844755	0.11275609	0.094843901	0.104364055	0.092958147	0.090856091	0.086319162	0.094846024
q<0.05	NaN	0.001519777	NaN	0.014095837	0.005790575	0.003252033	0.00660424	0.001317694
q<0.01	NaN	0.001519777	NaN	NaN	NaN	NaN	NaN	0.001317694
				channel 3				
Δ WTC	0.025519419	-0.001248873	0.007870212	0.011606211	0.008672769	0.007652393	0.002685346	0.005117751
SEM	0.024803864	0.024690311	0.023322281	0.0233783	0.024014216	0.023644776	0.022521179	0.021435182
tStat	1.028848532	-0.05058151	0.337454627	0.496452296	0.361151468	0.323639897	0.119236476	0.238754722
DF	191	143	143	95	95	95	47	47
pValues	0.304851981	0.959729581	0.736269033	0.620722125	0.71878813	0.746922068	0.905596211	0.81233378
Lower	-0.02340526	-0.050054018	-0.03823076	-0.034805583	-0.039001477	-0.039288422	-0.042621421	-0.038004273
Upper	0.074444099	0.047556272	0.053971183	0.058018005	0.056347015	0.054593208	0.047992113	0.048239775
q<0.05	NaN	NaN	NaN	NaN	NaN	NaN	NaN	NaN
q<0.01	NaN	NaN	NaN	NaN	NaN	NaN	NaN	NaN
				channel 4				
Δ WTC	0.019567169	0.007373769	0.013125996	0.011401642	0.011688431	0.016719011	0.007288641	0.010462282
SEM	0.020549484	0.019168525	0.021543072	0.020028217	0.017078939	0.017646065	0.017035827	0.017137019
tStat	0.952197586	0.38468105	0.609290793	0.569278936	0.684376855	0.947463974	0.427841929	0.610507707
DF	183	137	137	91	91	91	45	45
pValues	0.342252748	0.701070237	0.543340809	0.570569379	0.495476503	0.345911651	0.670806864	0.544596783
Lower	-0.020977208	-0.030530672	-0.029473948	-0.028381951	-0.022236784	-0.018332729	-0.027023276	-0.024053446
Upper	0.060111547	0.045278209	0.055725939	0.051185235	0.045613645	0.05177075	0.041600558	0.044978011
q<0.05	NaN	NaN	NaN	NaN	NaN	NaN	NaN	NaN
q<0.01	NaN	NaN	NaN	NaN	NaN	NaN	NaN	NaN
				channel 5				
Δ WTC	0.074509212	0.062393178	0.068455715	0.070104593	0.067753566	0.064035795	0.060428201	0.058735332
SEM	0.018643383	0.021086063	0.016011714	0.015532237	0.016854298	0.019254038	0.017428223	0.017906735
tStat	3.996550035	2.958977204	4.275352096	4.513489806	4.019957879	3.325837204	3.467261188	3.280069323
DF	207	155	155	103	103	103	51	51
pValues	8.94E-05	0.003571259	3.32E-05	1.70E-05	0.000111027	0.001222396	0.001076551	0.001874756
Lower	0.037753962	0.020740042	0.036826381	0.039300065	0.034327042	0.025849952	0.025439584	0.022786062
Upper	0.111264462	0.104046315	0.100085048	0.100909122	0.10118009	0.102221638	0.095416819	0.094684603
q<0.05	8.94E-05	0.003571259	3.32E-05	1.70E-05	0.000111027	0.001222396	0.001076551	0.001874756
q<0.01	8.94E-05	NaN	3.32E-05	1.70E-05	0.000111027	0.001222396	0.001076551	NaN

	50 s	60 s	70 s	80 s	90 s	100 s	complete	offline
				channel 6				
Δ WTC	0.026241689	0.016512164	0.011821303	0.013796569	0.012797091	0.007911195	0.007353582	0.012504651
SEM	0.021229576	0.02173859	0.018631176	0.019339029	0.019878902	0.020563261	0.019411998	0.019221724
tStat	1.236091068	0.759578458	0.63449044	0.713405467	0.643752398	0.384724721	0.378816312	0.650547817
DF	199	149	149	99	99	99	49	49
pValues	0.217881752	0.448706683	0.526733796	0.477273271	0.521224555	0.70126661	0.706460779	0.518377334
Lower	-0.015622112	-0.026443575	-0.024994147	-0.02457626	-0.026646963	-0.032890776	-0.031656289	-0.02612285
Upper	0.068105489	0.059467904	0.048636753	0.052169397	0.052241145	0.048713165	0.046363452	0.051132151
q<0.05	NaN	NaN	NaN	NaN	NaN	NaN	NaN	NaN
q<0.01	NaN	NaN	NaN	NaN	NaN	NaN	NaN	NaN
				channel 7				
Δ WTC	0.03740839	0.021764313	0.037169977	0.030536755	0.018279071	0.020671019	0.019546651	0.024156455
SEM	0.019520427	0.017998823	0.018022862	0.016774301	0.016194376	0.015894755	0.019358585	0.019735293
tStat	1.916371562	1.209207575	2.062379229	1.820448782	1.128729539	1.300493099	1.009714874	1.224023092
DF	207	155	155	103	103	103	51	51
pValues	0.056695245	0.228424328	0.040840729	0.071594402	0.26163406	0.196334319	0.317398598	0.22656817
Lower	-0.001075943	-0.01379033	0.001567847	-0.002731114	-0.013838655	-0.010852479	-0.01931733	-0.0154638
Upper	0.075892724	0.057318955	0.072772107	0.063804625	0.050396797	0.052194517	0.058410631	0.063776709
q<0.05	NaN	NaN	NaN	NaN	NaN	NaN	NaN	NaN
q<0.01	NaN	NaN	NaN	NaN	NaN	NaN	NaN	NaN
				channel 8				
Δ WTC	0.04814021	0.030880726	0.026700067	0.030842379	0.023681462	0.021242788	0.023426022	0.030997532
SEM	0.023514928	0.023293016	0.023511618	0.02247388	0.022274109	0.020789934	0.019749845	0.019435005
tStat	2.047219105	1.325750445	1.135611661	1.372365578	1.063183326	1.021782324	1.186137014	1.59493307
DF	199	149	149	99	99	99	49	49
pValues	0.041950699	0.186951326	0.257942482	0.173051005	0.290285311	0.309374325	0.241287941	0.117157723
Lower	0.001769794	-0.01514658	-0.019759199	-0.013750674	-0.020515204	-0.020008953	-0.016262777	-0.008058573
Upper	0.094510627	0.076908033	0.073159334	0.075435432	0.067878127	0.062494528	0.06311482	0.070053637
q<0.05	NaN	NaN	NaN	NaN	NaN	NaN	NaN	NaN
q<0.01	NaN	NaN	NaN	NaN	NaN	NaN	NaN	NaN
				channel 9				
Δ WTC	0.059852737	0.047245465	0.051393737	0.048311908	0.04130705	0.041476423	0.041823017	0.051118105
SEM	0.023413101	0.024050156	0.022344732	0.023369745	0.024584672	0.024035079	0.022115425	0.022158987
tStat	2.556378072	1.96445568	2.300038194	2.067284336	1.680195241	1.725661993	1.891124294	2.306879145
DF	191	143	143	95	95	95	47	47
pValues	0.011354306	0.051415977	0.022893149	0.041426973	0.09620494	0.087659547	0.064780139	0.025516067
Lower	0.013671286	-0.00029429	0.00722508	0.001917098	-0.007499696	-0.006239242	-0.002667479	0.006539973
Upper	0.106034188	0.094785221	0.095562395	0.094706718	0.090113795	0.089192088	0.086313513	0.095696237
q<0.05	0.011354306	NaN	NaN	NaN	NaN	NaN	NaN	NaN
q<0.01	NaN	NaN	NaN	NaN	NaN	NaN	NaN	NaN
				channel 10				
Δ WTC	0.046538818	0.044238755	0.044041549	0.03501105	0.032450541	0.033580219	0.032517727	0.037572807
SEM	0.015556298	0.015403502	0.017896688	0.019188713	0.018566329	0.017713689	0.019514913	0.020286759
tStat	2.99163832	2.87199343	2.460877001	1.824564728	1.747816719	1.895721356	1.666301425	1.852085183
DF	191	143	143	95	95	95	47	47
pValues	0.003140894	0.004700081	0.01505036	0.071209883	0.083726734	0.061036534	0.102305299	0.070300289
Lower	0.015854611	0.013790774	0.008665305	-0.003083363	-0.004408283	-0.0015859	-0.006741214	-0.003238889
Upper	0.077223025	0.074686737	0.079417793	0.073105462	0.069309365	0.068746337	0.071776669	0.078384502
q<0.05	0.003140894	0.004700081	0.01505036	NaN	NaN	NaN	NaN	NaN
q<0.01	NaN	NaN	NaN	NaN	NaN	NaN	NaN	NaN

Supplementary Table 2: Statistical results of the generalized linear mixed-effects model for the RPS data set for HbR.

	50 s	60 s	70 s	80 s	90 s	100 s	complete	offline
	channel 11							
Δ WTC	0.082768918	0.060012582	0.047811659	0.053864009	0.061239331	0.062509846	0.052271827	0.055137169
SEM	0.020485112	0.020623483	0.023135665	0.022027069	0.019939908	0.018491576	0.01981635	0.019043444
tStat	4.040442482	2.909915019	2.066578123	2.445355233	3.071194281	3.380449881	2.637813003	2.895335905
DF	199	149	149	99	99	99	49	49
pValues	7.62E-05	0.004169963	0.040504832	0.016237046	0.002752748	0.001036805	0.011151522	0.005642978
Lower	0.042373167	0.019260309	0.00209528	0.010157525	0.021674228	0.025818547	0.01244938	0.016867934
Upper	0.123164669	0.100764856	0.093528038	0.097570493	0.100804435	0.099201144	0.092094274	0.093406403
q<0.05	7.62E-05	0.004169963	NaN	0.016237046	0.002752748	0.001036805	0.011151522	0.005642978
q<0.01	7.62E-05	NaN	NaN	NaN	NaN	0.001036805	NaN	NaN
	channel 12							
Δ WTC	0.064374153	0.040702728	0.036799809	0.045428408	0.038684851	0.04063257	0.034815619	0.042725505
SEM	0.019766508	0.018882575	0.018463199	0.021348451	0.019844749	0.019160133	0.018267695	0.017797602
tStat	3.25672864	2.155570788	1.993143685	2.127948648	1.949374635	2.120683116	1.905857228	2.400632671
DF	191	143	143	95	95	95	47	47
pValues	0.001333623	0.032790666	0.048148458	0.035929884	0.054199889	0.036553085	0.062795022	0.020378091
Lower	0.025385467	0.003377688	0.000303747	0.003046374	-0.000711958	0.002594897	-0.001934244	0.006921348
Upper	0.103362838	0.078027767	0.07329587	0.087810442	0.078081659	0.078670242	0.071565482	0.078529662
q<0.05	0.001333623	NaN	NaN	NaN	NaN	NaN	NaN	NaN
q<0.01	0.001333623	NaN	NaN	NaN	NaN	NaN	NaN	NaN
	channel 13							
Δ WTC	0.097351474	0.086742072	0.082008575	0.084438047	0.092295862	0.087077726	0.075629286	0.081947376
SEM	0.019968545	0.02404461	0.024403223	0.026580415	0.024552056	0.023224376	0.022321881	0.022013504
tStat	4.875241312	3.607547429	3.360563232	3.176701678	3.75919078	3.749410731	3.388123318	3.722595798
DF	199	149	149	99	99	99	49	49
pValues	2.22E-06	0.000421229	0.000988161	0.001986933	0.00028846	0.000298479	0.001396227	0.000509023
Lower	0.057974372	0.039229605	0.033787484	0.031696738	0.043579257	0.040995525	0.030771786	0.037709584
Upper	0.136728577	0.134254539	0.130229665	0.137179357	0.141012468	0.133159928	0.120486786	0.126185168
q<0.05	2.22E-06	0.000421229	0.000988161	0.001986933	0.00028846	0.000298479	0.001396227	0.000509023
q<0.01	2.22E-06	0.000421229	0.000988161	NaN	0.00028846	0.000298479	0.001396227	0.000509023
	channel 14							
Δ WTC	0.064964087	0.086221684	0.073171303	0.067213377	0.059928543	0.061437098	0.060755124	0.051584323
SEM	0.027631688	0.025625942	0.026864625	0.029108843	0.027649324	0.026666341	0.025263108	0.025250388
tStat	2.351071954	3.364624965	2.723704601	2.309036402	2.167450553	2.303919329	2.404895082	2.042912077
DF	191	143	143	95	95	95	47	47
pValues	0.019738737	0.000984156	0.007260339	0.023108871	0.032700226	0.023406713	0.020168343	0.046691467
Lower	0.010461632	0.035567085	0.020068208	0.009425018	0.005037694	0.008497718	0.009932306	0.000787094
Upper	0.119466542	0.136876284	0.126274397	0.125001736	0.114819392	0.114376478	0.111577942	0.102381551
q<0.05	NaN	0.000984156	0.007260339	NaN	NaN	NaN	NaN	NaN
q<0.01	NaN	0.000984156	NaN	NaN	NaN	NaN	NaN	NaN
	channel 15							
Δ WTC	0.075509455	0.070522286	0.0715566	0.057264112	0.055510506	0.058806343	0.057059534	0.060933778
SEM	0.018541191	0.021860828	0.020216457	0.019387125	0.019432214	0.019051082	0.019426457	0.020289331
tStat	4.072524483	3.225965953	3.539522259	2.953718644	2.856622796	3.086771836	2.937207359	3.003242304
DF	199	149	149	99	99	99	49	49
pValues	6.71E-05	0.001543461	0.000535077	0.003922397	0.00521842	0.002624626	0.005034623	0.004198055
Lower	0.038947032	0.027325002	0.031608614	0.01879585	0.016952777	0.021004864	0.018020606	0.02016084
Upper	0.112071879	0.113719569	0.111504586	0.095732374	0.094068234	0.096607823	0.096098461	0.101706716
q<0.05	6.71E-05	0.001543461	0.000535077	0.003922397	0.00521842	0.002624626	0.005034623	0.004198055
q<0.01	6.71E-05	0.001543461	0.000535077	NaN	NaN	NaN	NaN	NaN

	50 s	60 s	70 s	80 s	90 s	100 s	complete	offline
	channel 16							
Δ WTC	0.044883538	0.03945439	0.030977688	0.020834813	0.027073559	0.024913649	0.02560171	0.025566624
SEM	0.021957438	0.022582094	0.020601804	0.022476637	0.021357985	0.022023429	0.020388889	0.019939193
tStat	2.04411547	1.747153769	1.503639611	0.926954186	1.267608256	1.131233895	1.25566969	1.282229647
DF	207	155	155	103	103	103	51	51
pValues	0.0422083	0.082591268	0.134708993	0.356117295	0.207796105	0.260583684	0.21495825	0.205560031
Lower	0.001594661	-0.005153987	-0.00971885	-0.023742295	-0.015284968	-0.018764628	-0.015330693	-0.014462976
Upper	0.088172415	0.084062767	0.071674227	0.06541192	0.069432085	0.068591926	0.066534113	0.065596223
q<0.05	NaN	NaN	NaN	NaN	NaN	NaN	NaN	NaN
q<0.01	NaN	NaN	NaN	NaN	NaN	NaN	NaN	NaN

Continuation of Supplementary Table 2
